# Reprogramming Exosomes to Escape from Immune Surveillance for Mitochondrial Protection in Hepatic Ischemia-Reperfusion Injury

**DOI:** 10.7150/thno.88061

**Published:** 2024-01-01

**Authors:** Shanshan Liu, Xinyu Xiao, La Zhang, Jianwei Wang, Wei Zhao, Haichuan Liu, Rui Liao, Zhi Li, Mengxia Xu, Jiao Guo, Baoyong Zhou, Chengyou Du, Qiling Peng, Ning Jiang

**Affiliations:** 1 School of Basic Medical Science, Chongqing Medical University, Chongqing 400016, P. R. China.; 2 Department of Hepatobiliary Surgery, the First Affiliated Hospital of Chongqing Medical University, Chongqing 400016, P. R. China.; 3 Traditional Chinese Medicine Hospital of Bijie City, Guizhou province, 551700, People's Republic of China.; 4 Bijie Municipal Health Bureau, Guizhou province, 551700, People's Republic of China.; 5 Department of Pathology, Chongqing Medical University, Chongqing 400016, P. R. China.; 6 Molecular Medicine Diagnostic and Testing Center, Chongqing Medical University, Chongqing 400016, P. R. China.; 7 Department of Pathology, the First Affiliated Hospital of Chongqing Medical University, Chongqing 400016, P. R. China.; 8 Department of Plastic and Maxillofacial Surgery, The Second Affiliated Hospital of Chongqing Medical University, Chongqing 400016, P. R. China.

**Keywords:** Exosomes, Hepatic ischemia/reperfusion injury, Macrophages, CD47, Mitochondria, Cyclosporin A

## Abstract

**Background:** Therapeutic interventions such as synthetic drugs and microRNA (miR) modulators have created opportunities for mitigating hepatic ischemia/reperfusion injury (HIRI) by alleviating mitochondrial dysfunction. However, delivering multi-therapeutic ingredients with low toxicity to hepatocytes still lags behind its development.

**Methods:** In this study, we endowed exosomes with delivery function to concentrate on hepatocytes for multidimensionally halting mitochondria dysfunction during HIRI. Concretely, exosomes were reprogrammed with a transmembrane protein CD47, which acted as a “camouflage cloak” to mimic the “don't eat me” mechanism to escape from immune surveillance. Besides, HuR was engineered bridging to the membrane by fusing with CD47 and located in the cytoplasm for miR loading.

**Results:** This strategy successfully delivered dual payloads to hepatocytes and efficiently protected mitochondria by inhibiting the opening of mitochondrial permeability transition pore (mPTP) and upregulating mitochondrial transcription factor A (TFAM), respectively.

**Conclusions:** The reprogramming of exosomes with CD47 and HuR for targeted delivery of CsA and miR inhibitors represents a promising therapeutic strategy for addressing HIRI. This approach shows potential for safe and effective clinical applications in the treatment of HIRI.

## Introduction

Hepatic ischemia/reperfusion injury (HIRI), an inevitable pathological insult during liver surgery such as liver resection and transplantation, presents one of the major causes of perioperative morbidity and mortality 1. HIRI is a two-phase event, in which cell damage initially results from oxygen deficiency by insufficient blood flow and is exacerbated upon restoration of oxygen supply 2. As a characteristic of the ischemic phase, the damage of mitochondria which are particularly sensitive to hypoxia, embodies in oxidative phosphorylation decline, ATP depletion, and metabolite accumulation 3. Once blood supply is restored, excessive metabolites, especially reactive oxygen species (ROS), intensify mitochondrial dysfunction, as manifested by the depolarization of membrane potential, opening of mitochondrial permeability transition pore (mPTP), and damage of mitochondrial DNA (mtDNA) 3. Accompanied by mitochondrial damage aggravating, pro-apoptotic factors such as cytochrome C (CytC) are released, ultimately leading to hepatocyte death and triggering a cascade of HIRI events. In general, mitochondria are critical instigators and later effectors of HIRI, making the degree of mitochondrial dysfunction directly proportional to the severity of HIRI. Accordingly, the critical importance of mitochondria in HIRI highlights mitochondria-targeted approaches as potential therapeutic avenues for future treatments.

Accumulated evidence has revealed the crucial role of mitochondria as therapeutic targets in ameliorating HIRI 4,5. Strategies for mitigating mitochondrial damage generally concentrate on modulating mitochondrial morphology, reversing mitochondrial respiratory chain dysfunction, especially suppressing the opening of mPTP and maintaining mitochondrial homeostasis 6. Previous studies have confirmed the therapeutic efficacy of cyclosporin A (CsA), a polypeptide, which indirectly interferes with mPTP opening by competitively inhibiting cyclophilin D (CypD), a key regulator of mPTP 6,7. Besides, microRNA (miR)-based therapy has demonstrated potential in maintaining mitochondrial homeostasis by regulating the expression of mitochondrial regulator proteins, such as mitochondrial transcription factor A (TFAM) 8,9. TFAM plays a crucial role in sustaining mtDNA stability, and an increase of its expression has been shown to relieve HIRI significantly 10. Given the complexity of mitochondrial damage, carrier systems to co-deliver therapeutic ingredients are potential approaches to effectively halt the progress of HIRI. However, the inevitable side effects of conventional carrier systems are required to be addressed, which arise from insufficient targeting precision, low solubility and stability, and poor biocompatibility 11. Hence, developing an innovative delivery system for CsA and miR-modulators, which inhibit CypD and upregulate TFAM, respectively, to alleviate HIRI is paramount.

Recently, nanoparticle (NP)-based therapeutics have depicted prospective approaches in alleviating HIRI, such as Ceria NPs 12 and platinum NPs 13. However, as foreign particles, NPs still exist some issues in toxicity, efficacy, and stability 14. Gratifyingly, as one of the small extracellular vesicles with remarkable biocompatibility, stability, and favorable safety profile, exosomes (Exos) have emerged as promising drug delivery vehicles compared with traditional ones 15. Owing to their unique “tropism” capability, Exos could target and engage with cells from their parental tissue selectively, thereby enhancing the precision of therapeutic delivery 16. Nevertheless, macrophages still craft a hurdle to the effectiveness of Exos in delivering to hepatocytes 17. Therefore, the ideal delivery vehicle should possess the capability to evade immune surveillance of macrophages, particularly Kupffer cells.

**Scheme 1:**
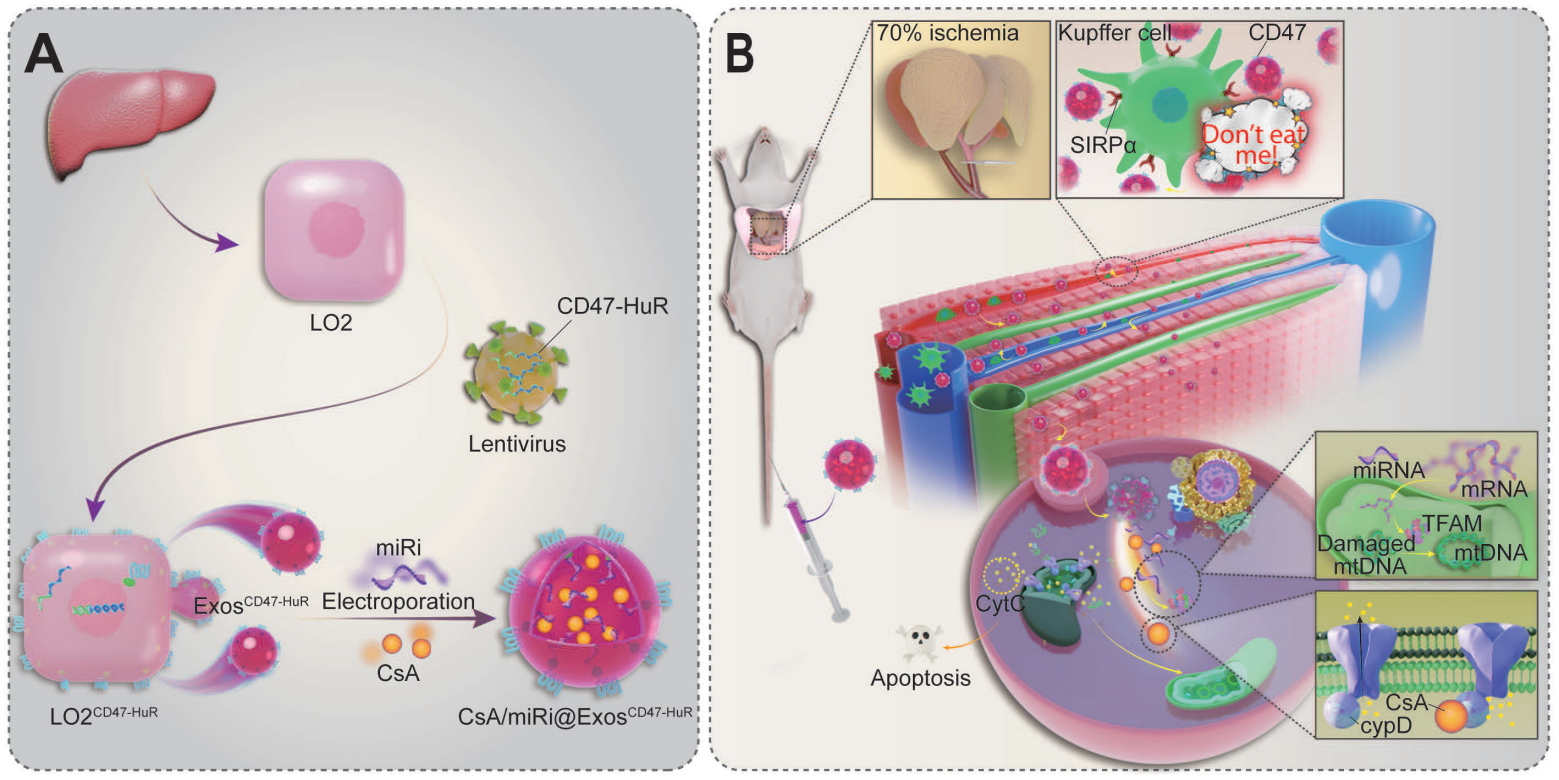
** Scheme of reprogrammed exosomes for alleviating hepatic ischemia/reperfusion injury via mitochondria protection.** (**A**) The human liver cells (LO2) were functionalized with CD47-HuR fusion protein (LO2^CD47-HuR^). The Cyclosporin A (CsA) and mitochondrial transcription factor A (TFAM) targeting microRNA inhibitor (miRi) were loaded into LO2^CD47-HuR^-derived exosomes by electroporation to prepare CsA/miRi@Exos^CD47-HuR^. (**B**) The mechanism of CsA/miRi@Exos^CD47-HuR^ to alleviate HIRI by inhibiting the opening of mitochondrial permeability transition pore (mPTP) and upregulating TFAM.

To accommodate the demands of escaping from immune surveillance and co-loading CsA and miR-modulators with fewer side effects, inspired by the ability of CD47 expressed on tumor cells emitting a “don't eat me” signal to macrophages and human antigen R (HuR) interacting with AU-rich elements (AREs) to bind target RNA 18,19, we established a nimbly reprogrammed Exos wearing the CD47 “camouflage cloak” and grafting the “AREs-magnetism” HuR. In detail, reprogrammed Exos overexpressed CD47-HuR and efficiently delivered CsA and miR inhibitor (miRi) targeting TFAM to hepatocytes for assuaging HIRI, referred to as CsA/miRi@Exos^CD47-HuR^, as shown in Scheme 1. In summary, our study introduces an innovative method of utilizing reprogrammed Exos as efficient drug carriers for both pharmaceutical ingredients and RNA molecules, which seeds hope for HIRI therapy against mitochondrial dysfunction.

## Results

### Exos reprogrammed with the CD47-HuR fusion protein and the identification of miR modulators

**Figure 1:**
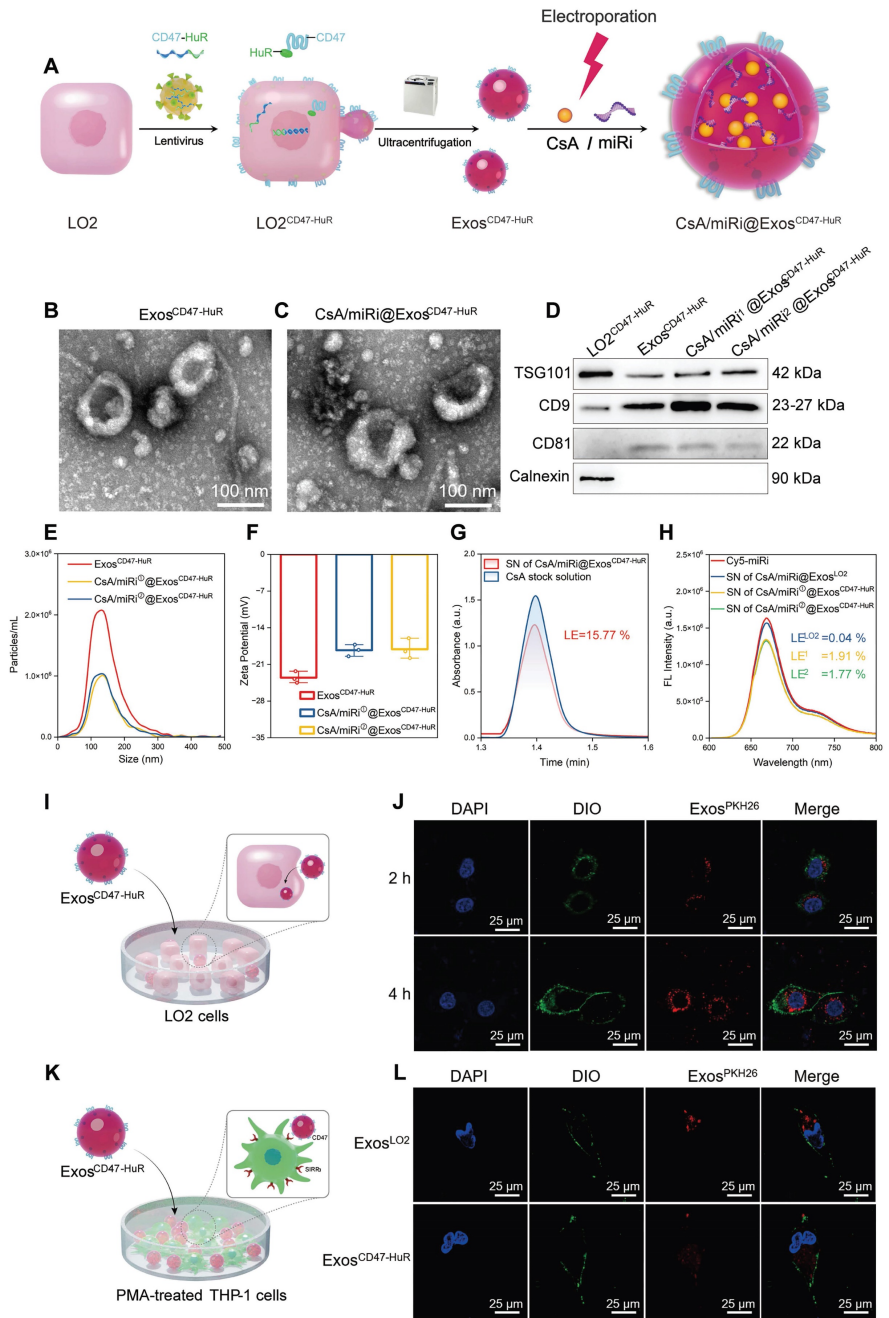
**Preparation and molecular characterization of reprogrammed Exos.** (**A**) Schematic illustration of CsA/miRi@Exos^CD47-HuR^ preparation. Transmission electron microscope (TEM) images of Exos^CD47-HuR^ isolated from lentivirus-infected LO2 (LO2^CD47-HuR^) cells (**B**) and CsA/miRi@Exos^CD47-HuR^ which were loaded with CsA and miRi simultaneously through electroporation (**C**). Scale bars: 100 nm. (**D**) Exosomal biomarkers were assessed using western blot analysis, proteins of LO2^CD47-HuR^ cells (lane 1), Exos^CD47-HuR^ (lane 2), CsA/miRi^①^@Exos^CD47-HuR^ (lane 3), and CsA/miRi^②^@Exos^CD47-HuR^ (lane 4). TSG101, CD9, and CD81 were Exo-specific biomarkers that enriched in Exos, while the endoplasmic reticulum marker, Calnexin, was devoid. (**E**) Particle size (nm) and concentration (particles/mL) of Exos were measured by nanoparticle tracking analysis (NTA). (**F**) Zeta potential (ξ) histogram of Exos^CD47-HuR^, CsA/miRi^①^@Exos^CD47-HuR^, CsA/miRi^②^@Exos^CD47-HuR^, respectively. (**G**) The absorbance of CsA stock solution and supernatant (SN) of CsA/miRi@Exos^CD47-HuR^ after electroporation and ultracentrifugation using high-performance liquid chromatography with ultraviolet detection (HPLC-UV) at a wavelength of 210 nm. Loading efficiency (LE) of CsA was quantified by HPLC-UV. The LE was calculated by the formula of (W_CsA_-W_free CsA_)/W_Exos_ × 100%, where W_CsA_ was the total weight of CsA, W_free CsA_ was the weight of unbounded CsA, and W_Exos_ was the weight of Exos. (**H**) Fluorescent spectrum analyses of aqueous solution of Cy5-labeled miRi, SN of CsA/miRi@Exos^LO2^, SN of CsA/miRi^①^@Exos^CD47-HuR^, and SN of CsA/miRi^②^@Exos^CD47-HuR^ after electroporation and ultracentrifugation, with excitation at 650 nm and emission at 670 nm. The LE was calculated by: (W_miRi_-W_free miRi_)/W_Exos_ × 100%, where W_miRi_ was the weight of total miRi, W_free miRi_ was the weight of unbounded miRi, and W_Exos_ was the weight of Exos. (**I**) Schematic illustration of Exos^CD47-HuR^ being internalized by LO2 cells. (**J**) Confocal images display the *in vitro* uptake of Exos^CD47-HuR^ by LO2 cells within 4 h. Nuclei were stained with 4', 6-diamidino-2-phenylindole (DAPI, blue), Exos were labeled with PKH-26 (red), and cell membranes were stained with 3,3'-dioctadecyloxacarbocyanine perchlorate (DIO, green). Scale bar: 25 µm. (**K**) Schematic illustration of Exos^CD47-HuR^ escaping phagocytosis from THP-1-M cells. (**L**) Exos functionalized with CD47-HuR have a reduced likelihood of being internalized by THP-1-M cells. Nuclei were stained with DAPI (blue), Exos were labeled with PKH-26 (red), and cell membranes were stained with DIO (green). THP-1-M cells were differentiated with 100 ng/mL Phorbol-12-myristate-13-acetate (PMA) for 48 h to differentiate macrophages *in vitro*. Scale bar: 25 µm. miRi^①^ represents hsa-miR-590-3p inhibitor, miRi^②^ represents mmu-miR-7057-3p inhibitor.

Inspired by the “camouflage cloak” function of CD47 18 to evade immune surveillance and the capacity of HuR to bind AREs 19, a functionalized fusion protein was successfully developed using advanced genetic engineering techniques (Figure 1A). Specifically, the target fusion gene CD47-HuR was integrated into LO2 cells via lentiviral infection, leading to overexpression of the CD47-HuR fusion protein in LO2 cells, which were referred to as LO2^CD47-HuR^ cells (Figure S1A and B). Exos secreted from LO2 or LO2^CD47-HuR^ cells were extracted by ultracentrifugation, with Exos^CD47-HuR^ inheriting overexpression of the CD47-HuR fusion protein (Figure S1C), signifying the successful integration of CD47-HuR onto Exos. To fully harness the potential of HuR, the TargetScan database 20 was searched for miRs with a high content of AREs that could regulate TFAM expression in *Homo sapiens* (human, hsa) or *Mus musculus* (mouse, mmu). Subsequently, hsa-miR-590-3p and mmu-miR-7057-3p were identified, as shown in Figure S2A and E. In addition, western blot and quantitative real-time polymerase chain reaction (qRT-PCR) were performed to validate whether the miR modulators possessed this functionality in LO2 and NCTC-1469 cells (Figure S2B-D and F-H). As expected, our results demonstrated that the miR inhibitor (miRi) could significantly upregulate TFAM expression. Thus, the reprogrammed Exos^CD47-HuR^ were successfully fabricated, and miR modulators that could increase TFAM expression were obtained.

### Characterization and antiphagocytic function of CsA/miRi@Exos^CD47-HuR^ *in vitro*

After extracting Exos^CD47-HuR^ from donor cells (LO2^CD47-HuR^), CsA and miRi were concurrently incorporated into Exos via electroporation (Figure 1A). Subsequently, transmission electron microscopy (TEM) revealed the characteristic round-shaped morphology of Exos^CD47-HuR^ (Figure 1B and C), while western blot showed the expression of Exo-specific markers (TSG101, CD9, CD81) 21 and an endoplasmic reticulum marker (Calnexin) 22 (Figure 1D). Nanoparticle tracking analysis (NTA) of Exos^CD47-HuR^ revealed an average size of 142 nm at a concentration of approximately 2.0 × 10^6^ particles/mL (Figure 1E), and the zeta potential (ξ) was negative (- 23.53 mV) (Figure 1F). Notably, electroporation did not affect their morphology (Figure 1C), protein expression (Figure 1D), average size (Figure 1E), or zeta potential (ξ) (Figure 1F). Furthermore, the loading efficiency (LE) of CsA in Exos^CD47-HuR^ was determined to be 15.77% by high-performance liquid chromatography with ultraviolet detection (HPLC-UV) (Figure 1G), while the LE of miRi altered from 0.04% in Exos^LO2^ to approximately 2.0% in Exos^CD47-HuR^, as measured by a fluorescence spectrophotometer (Figure 1H), indicating that the overexpression of CD47-HuR increased the LE of miRi into Exos. Having fabricated reprogrammed Exos, *in vitro* experiments were conducted to assess their internalization. Confocal laser scanning microscopy (CLSM) confirmed the cellular uptake of Exos (labeled with the fluorescent dye PKH26) after incubation with LO2 cells or phorbol-12-myristate-13-acetate (PMA) treated THP-1 cells (THP-1-M). Exos^CD47-HuR^ were effectively internalized by LO2 cells rather than THP-1-M cells after 4 h of incubation (Figure 1I-L, Fig S3), and the uptake efficiency of Exos^LO2^ without CD47-HuR overexpression was higher in THP-1-M cells, suggesting that Exos^CD47-HuR^ could evade immune surveillance. The *in vitro* release assay of CsA and miRi from Exos^CD47-HuR^ indicated that 50% of CsA was released within the initial 12 h, while 80% of it was released within 24 h, whereas 50% of miRi was released within the first 24 h, with 80% of miRi being released within 36 h (Figure S4). Under these circumstances, CsA/miRi@Exos^CD47-HuR^ with high loading of dual cargos and high distribution in target cells could be used for further *in vitro* and *in vivo* investigations to verify therapeutic benefits.

### Antiapoptosis effects of CsA/miRi@Exos^CD47-HuR^ by protecting mitochondria *in vitro*

**Figure 2:**
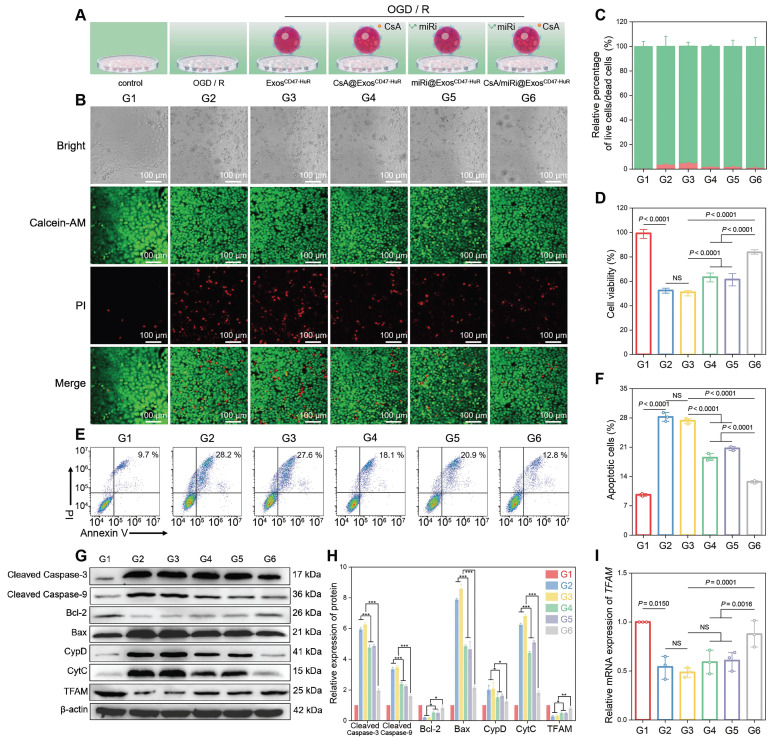
**Therapeutic effects and anti-apoptosis mechanism of drug-loaded Exos^CD47-HuR^ *in vitro*.** (**A**) Schematic illustration of group design. Groups were designed as: normal LO2 cells (G1); LO2 cells after OGD/R (G2); LO2 cells pretreated with Exos^CD47-HuR^ before OGD/R (G3); LO2 cells pretreated with CsA@Exos^CD47-HuR^ before OGD/R (G4); LO2 cells pretreated with miRi@Exos^CD47-HuR^ before OGD/R (G5); LO2 cells pretreated with CsA/miRi@Exos^CD47-HuR^ before OGD/R (G6). miRi refers to hsa-miR-590-3p inhibitor. (**B**) Confocal laser scanning microscope (CLSM) images of LO2 cells co-stained with calcein AM/PI after pretreatments and OGD/R. Green: live cells; Red: dead cells. Scale bar: 100 μm. (**C**) Relative percentage of live and dead LO2 cells across different treatment groups, calculated by ImageJ. (**D**) Cell viability of LO2 cells after OGD/R declined to 50%, and recovered to 80% after pretreatment with CsA/miRi@Exos^CD47-HuR^. (**E**) LO2 cell apoptosis was analyzed using Annexin V/PI staining by flow cytometry, with quantitative analysis shown in (**F**). (**G**) Western blot of apoptosis-related protein and TFAM expression in LO2 cells. (**H**) Quantification of apoptosis-related protein and TFAM expression. (**I**) qRT-PCR analysis of the mRNA level of *TFAM* in LO2 cells after being treated with different formulations. Data are expressed as mean ± S.D. (n = 3), ***P < 0.001, **P < 0.01, *P < 0.05, NS means no difference (non-repeated ANOVA followed by Tukey's test).

Having established well-crafted Exos loaded with CsA and miRi, further studies were conducted to investigate whether CsA/miRi@Exos^CD47-HuR^ could alleviate the damage induced by oxygen-glucose deprivation/ reoxygenation (OGD/R) *in vitro*. First, OGD for 6 h and R for 6 h was selected to maximize the antiapoptosis effects, because the expression of apoptosis-related proteins (cleaved caspase 3, cleaved caspase 9, Bcl-2, Bax, CypD, and CytC) 23 and TFAM reached a plateau in LO2 cells (Figure S5). Furthermore, CsA/miRi@Exos^CD47-HuR^ and other treatments were incubated with LO2 cells for 24 h before OGD/R, as shown in Figure 2A. Afterward, CLSM was used to analyze LO2 cells stained with calcein AM and propidium iodide (PI) and effectively visualize live and dead cells (Figure 2B and C). Furthermore, the cell counting kit-8 assay revealed that less than 51.5% of cells survived following OGD/R, whereas over 83.7% of cells remained viable after pretreatment with CsA/miRi@Exos^CD47-HuR^ (Figure 2D, Figure S6D). More importantly, we observed a significant decrease in cell apoptosis after treatment with CsA/miRi@Exos^CD47-HuR^, indicating that these factors significantly maintained cell viability (Figure 2E and F, Figure S6A and C). Furthermore, the expression of apoptosis-related proteins was evaluated by western blot and revealed that CsA/miRi@Exos^CD47-HuR^ could robustly alleviate apoptosis, while CsA@Exos^CD47-HuR^ and miRi@Exos^CD47-HuR^ exerted less pronounced effects (Figure 2G and H). Overall, the antiapoptotic effects of CsA/miRi@Exos^CD47-HuR^ on the OGD/R model confirmed that among the different treatments, CsA/miRi@Exos^CD47-HuR^ provided optimal protection of LO2 cells.

**Figure 3:**
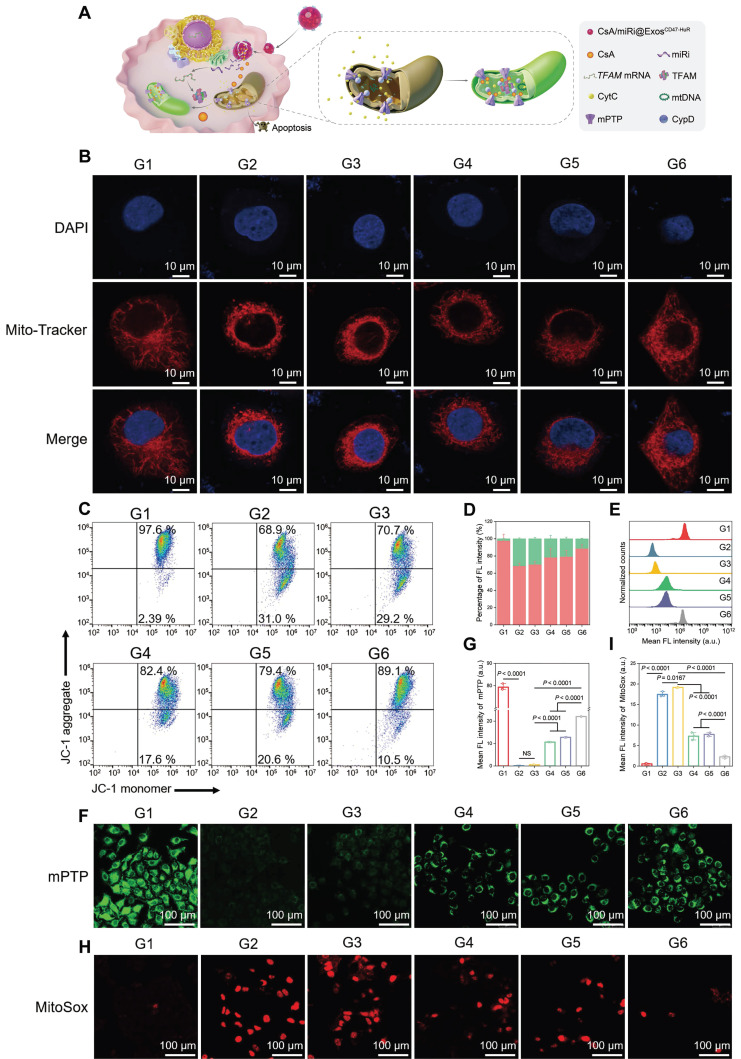
**Protective effects against mitochondrial dysfunction of drug-loaded Exos^CD47-HuR^ *in vitro*.** (**A**) Schematic illustration of CsA/miRi@Exos^CD47-HuR^ protecting mitochondria. (**B**) Representative CLSM images showing mitochondrial morphology stained with MitoTracker (red) and DAPI (blue). Scale bar: 10 μm. (**C**) Mitochondrial membrane potential (Δψm) detected by flow cytometry after stained with JC-1 dye. (**D**) The percentages of JC-1 aggregates (mitochondrial membrane potential intact, red) and JC-1 monomers (mitochondrial membrane potential lost, green) indicate the extent of mitochondrial-dependent apoptosis. The extent of mPTP opening was evaluated by calcein AM (green) staining and then detected by flow cytometry (**E**) and CLSM (**F**). Scale bar: 100 μm. (**G**) Quantification of the extent of mPTP opening from CLSM. (**H**) Representative confocal images of reactive oxygen species generated from mitochondria stained with MitoSox (red). Scale bar: 100 μm. (**I**) Quantitative analysis of MitoSox fluorescence intensity. Groups were designed as: normal LO2 cells (G1); LO2 cells after OGD/R (G2); LO2 cells pretreated with Exos^CD47-HuR^ before OGD/R (G3); LO2 cells pretreated with CsA@Exos^CD47-HuR^ before OGD/R (G4); LO2 cells pretreated with miRi@Exos^CD47-HuR^ before OGD/R (G5); LO2 cells pretreated with CsA/miRi@Exos^CD47-HuR^ before OGD/R (G6). miRi refers to hsa-miR-590-3p inhibitor. Data are expressed as mean ± S.D. (n = 3), NS means no difference (non-repeated ANOVA followed by Tukey's test).

As a classic pathway of programmed cell death, apoptosis can be triggered by the activation of the mitochondrial pathway 24. Concomitantly, TFAM, a key regulator of mitochondrial gene expression 25, was upregulated at the protein and mRNA levels after treatment with CsA/miRi@Exos^CD47-HuR^, as determined by western blot and qRT-PCR (Figure 2G-I). Therefore, we examined whether CsA/miRi@Exos^CD47-HuR^ exerted antiapoptotic effects by protecting mitochondria, as shown in Figure 3A. First, Mito Tracker (red) staining was used to monitor mitochondrial morphology in LO2 cells that were pretreated with CsA/miRi@Exos^CD47-HuR^ and other Exos after OGD/R (Figure 3B, Figure S6B). Consequently, CsA/miRi@Exos^CD47-HuR^ partially restored the filamentous network of mitochondria in LO2 cells, suggesting its potential to ameliorate mitochondrial damage by directly maintaining mitochondrial morphology. Subsequently, mitochondrial functional assays were performed to examine mitochondrial membrane potential (Δψm), mPTP opening, and mitochondrial ROS levels. Specifically, the change in Δψm was monitored by JC-1 staining, and aggregates represent healthy polarized mitochondria (red), whereas monomers (green) indicate unhealthy depolarized mitochondria 26. Flow cytometry and CLSM showed that OGD/R exhibited decreased JC-1 aggregates and increased JC-1 monomers, while CsA/miRi@Exos^CD47-HuR^ effectively restored Δψm (Figure 3C and D, Figure S7A and B). Concurrently, the extent of mPTP opening was assessed via a fluorescence assay kit, which revealed that CsA@Exos^CD47-HuR^, miRi@Exos^CD47-HuR^, and CsA/miRi@Exos^CD47-HuR^ could inhibit mPTP opening, as indicated by flow cytometry and CLSM (Figure 3E-G, Figure S7C). Moreover, representative CLSM images of MitoSox (red) which is a mitochondrial-specific ROS indicator, showed the same outcomes, as shown in Figure 3H and I. Accordingly, a significant amount of ROS (DCFH-DA probe) was found in the cytoplasm of LO2 cells after OGD/R, while incubating LO2 cells with CsA/miRi@Exos^CD47-HuR^ significantly reduced the fluorescence intensity, indicating the potent scavenging of ROS, as shown in Figure S7D and E. In summary, our results demonstrated a significant reduction in apoptosis, particularly through the alleviation of mitochondrial dysfunction after treatment with CsA/miRi@Exos^CD47-HuR^ in the OGD/R model, suggesting that the use of CsA/miRi@Exos^CD47-HuR^ might be a therapeutic strategy for mitigating OGD/R-induced cell damage.

### *In vivo* distribution and antiphagocytic capabilities of Exos^CD47-HuR^

**Figure 4:**
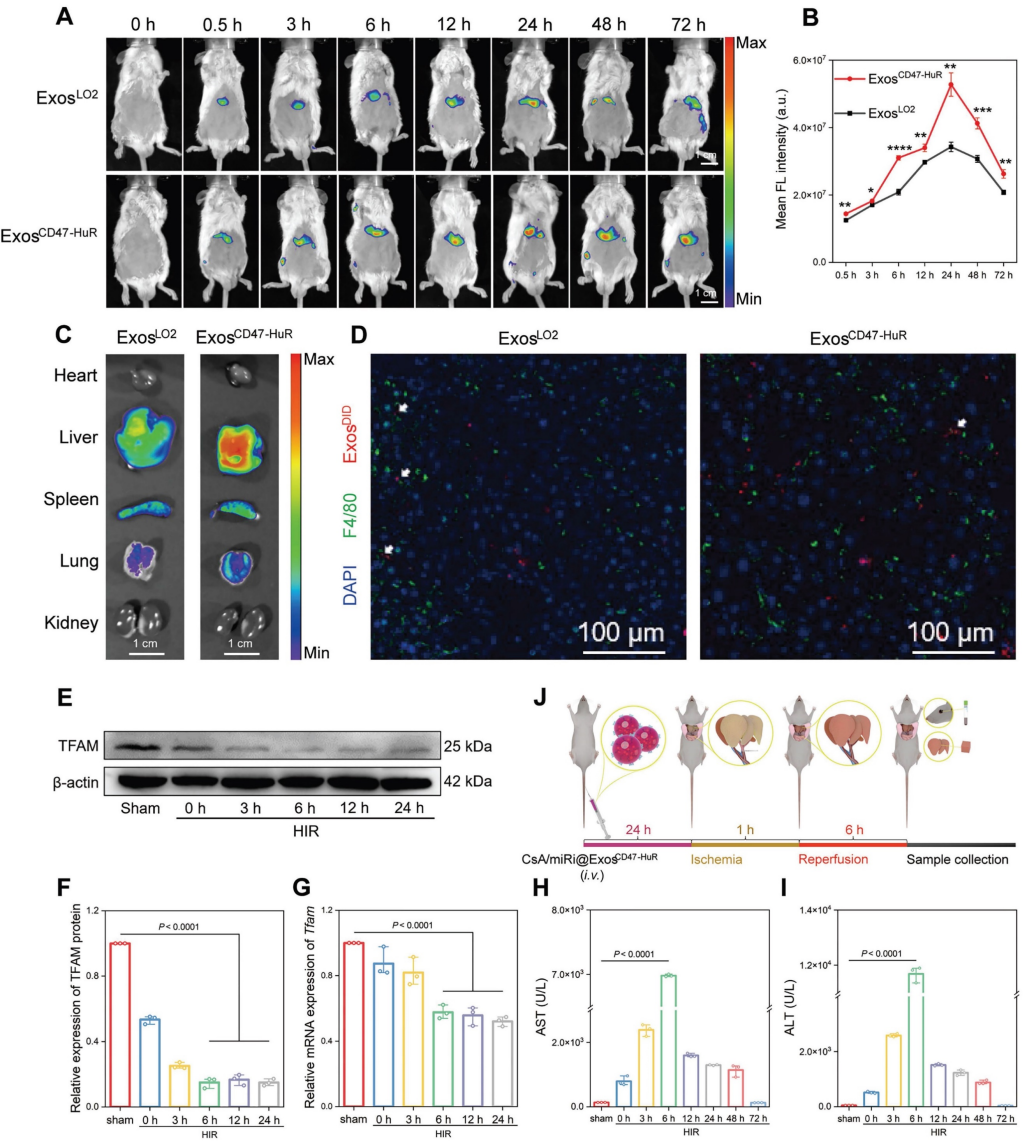
***In vivo* distribution and phagocytosis evasion of Exos^CD47-HuR^.** (**A**) *In vivo* fluorescence images show the accumulation of 1,1'-dioctadecyl-3,3,3',3'-tetramethylindotricarbocyanine iodide (DIR)-labeled Exos^LO2^ and Exos^CD47-HuR^ in the liver of BALB/c mice. (**B**) Quantification of fluorescence intensity in the liver at various time points, with the Exos^CD47-HuR^ exhibiting a superior accumulation than Exos^LO2^. Data are mean ± S.D. (n = 3), ***P < 0.001, **P < 0.01, *P < 0.05 (Student's t-test). (**C**) Tissue distribution analyses show that Exos^CD47-HuR^ is preferentially targeted to the liver. (**D**) Representative CLSM images show that Exos^CD47-HuR^ can optimize their phagocytosis evasion ability. Macrophages were labeled green with F4/80, Exos were labeled red with 1'-Dioctadecyl-3,3,3',3'-tetramethylindodicarbocyanine, 4-chlorobenzenesulfonate salt (DID), and nuclei were labeled blue with DAPI. (**E-G**) Protein and mRNA expression of TFAM at different reperfusion time points: 3 h, 6 h, 12 h, and 24 h, with the most significant decrease at 6 h. (**H, I**) Levels of aspartate transaminase (AST) and alanine transaminase (ALT) peaked at 6 h after reperfusion. (**J**) A step-wise overview of CsA/miRi@Exos^CD47-HuR^ injection, ischemia/reperfusion procedure, and follow-up sample collection for BALB/c mice. Data are expressed as mean ± S.D. (n = 3), (non-repeated ANOVA followed by Tukey's test).

The cytoprotective effects of CsA/miRi@Exos^CD47-HuR^ against apoptosis through the attenuation of mitochondrial dysfunction was validated *in vivo*. First, the drug delivery capability of reprogrammed Exos to hepatocytes was evaluated by injecting 1,1'-dioctadecyl-3,3,3',3'-tetramethylindotricarbocyanine iodide (DIR)-labeled Exos^LO2^ or Exos^CD47-HuR^, and the maximum fluorescence intensity was observed at 24 h postinjection (Figure 4A). Consistently, quantitative analysis showed that the amount of Exos^CD47-HuR^ in the liver was approximately 1.54-fold higher than that of Exos^LO2^ at 24 h postinjection (Figure 4B). Furthermore, the fluorescence intensity in the liver persisted even at 72 h postinjection. In contrast, there was a lower fluorescence intensity and earlier disappearance of Exos^LO2^ (Figure 4A), suggesting effective evasion of phagocytosis by Exos^CD47-HuR^. In addition, the *ex vivo* images showed a high level of fluorescence in liver tissue, particularly in the group that was injected with Exos^CD47-HuR^ (Figure 4C, right). To further examine this effect, mice were injected with 1,1'-dioctadecyl-3,3,3',3'-tetramethylindodicarbocyanine,4-chlorobenzenesulfonate salt (DID)-labeled Exos, liver tissues were collected at 24 h postinjection and macrophages were labeled by the macrophage marker F4/80 27. Consistent with the *in vitro* results, CLSM showed reduced uptake of Exos^CD47-HuR^ (red) by macrophages (green) (Figure 4D). Overall, Exos^CD47-HuR^ were more capable of phagocytic escape than Exos^LO2^, highlighting the superior ability of Exos^CD47-HuR^ with an extended half-life in liver tissue to deliver drugs precisely.

### Therapeutic efficacy and biosafety of CsA/miRi@Exos^CD47-HuR^ in the HIRI model

Exos^CD47-HuR^ can evade phagocytosis and persist for a long time in the livers of mice, suggesting that Exos^CD47-HuR^ can systematically deliver CsA and miRi to hepatocytes, which might exert protective effects on mitochondria *in vivo*. First, a model of 70% warm hepatic ischemia for 60 min was established according to a previous method 28, followed by reperfusion for 3, 6, 12, 24, 48, and 72 h to determine the optimal reperfusion time. Then, liver tissue and blood samples were prepared for protein or RNA extraction and biochemical analysis, and the results showed that TFAM protein and mRNA levels decreased with increasing reperfusion times (Figure 4E-G). Moreover, liver enzymes, such as aspartate aminotransferase (AST) and alanine aminotransferase (ALT), peaked at 6 h postreperfusion, suggesting maximal liver damage at this time point (Figure 4H and I). As a result, BALB/c mice were subjected to partial warm hepatic ischemia for 60 min followed by a 6 h of reperfusion in the HIRI model.

**Figure 5:**
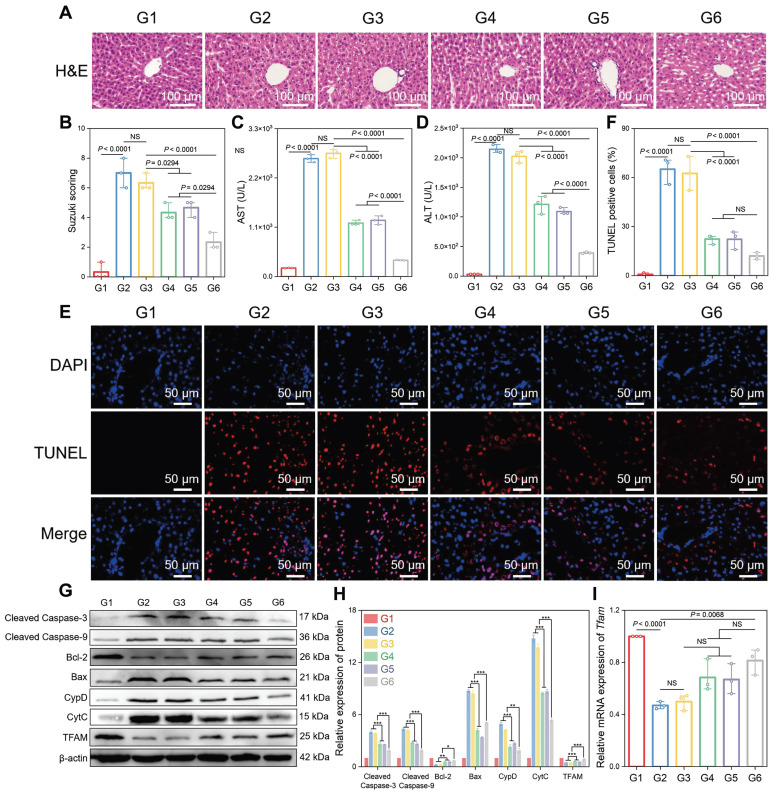
** Therapeutic efficacy of CsA/miRi@Exos^CD47-HuR^ *in vivo*.** (**A**) Hematoxylin & Eosin (H&E) staining of residual liver ischemia tissue with drug-loaded Exos^CD47-HuR^ restored liver injury. (**B**) Quantification of liver damage calculated by Suzuki score shows significant therapy efficiency. (**C**, **D**) Levels of AST and ALT with administration of different Exos (CsA@Exos^CD47-HuR^, miRi@Exos^CD47-HuR,^ or CsA/miRi@Exos^CD47-HuR^) decreased significantly. (**E**, **F**) Liver cell apoptosis by terminal deoxynucleotidyl transferase (TdT) dUTP nick end labeling (TUNEL) staining. (**G**, **H**) Apoptosis-related proteins and TFAM expression detected by western blot. (**I**) mRNA level of *Tfam* by qRT-PCR from liver tissue. Groups were designed as: sham (G1); BALB/c mice with HIRI (G2); BALB/c mice pretreated with Exos^CD47-HuR^ before HIRI (G3); BALB/c mice pretreated with CsA@Exos^CD47-HuR^ before HIRI (G4); BALB/c mice pretreated with miRi@Exos^CD47-HuR^ before HIRI (G5); BALB/c mice pretreated with CsA/miRi@Exos^CD47-HuR^ before HIRI (G6). miRi refers to mmu-miR-7057-3p inhibitor. Data are expressed as mean ± S.D. (n = 3), ***P < 0.001, **P < 0.01, *P < 0.05, NS means no difference (non-repeated ANOVA followed by Tukey's test).

Subsequently, the therapeutic potential of CsA/miRi@Exos^CD47-HuR^ was verified by injecting different groups of Exos into BALB/c mice 24 h before HIRI (Figure 4J). As expected, CsA/miRi@Exos^CD47-HuR^ exhibited significantly superior therapeutic efficacy compared with those in the other groups (Figure 5). Hematoxylin & Eosin (H&E) staining and the Suzuki score were used to visualize the morphology of the liver and quantify disordered organization, including congestion, cytoplasmic parenchymal vacuolization, and necrosis, in HIRI mice, as shown in Figure 5A and B. In addition, AST and ALT analyses showed that CsA/miRi@Exos^CD47-HuR^ could protect liver function (Figure 5C and D). Overall, treatment with drug-loaded Exos^CD47-HuR^ ameliorated the damage to ischemic liver regions with superior efficacy.

**Figure 6:**
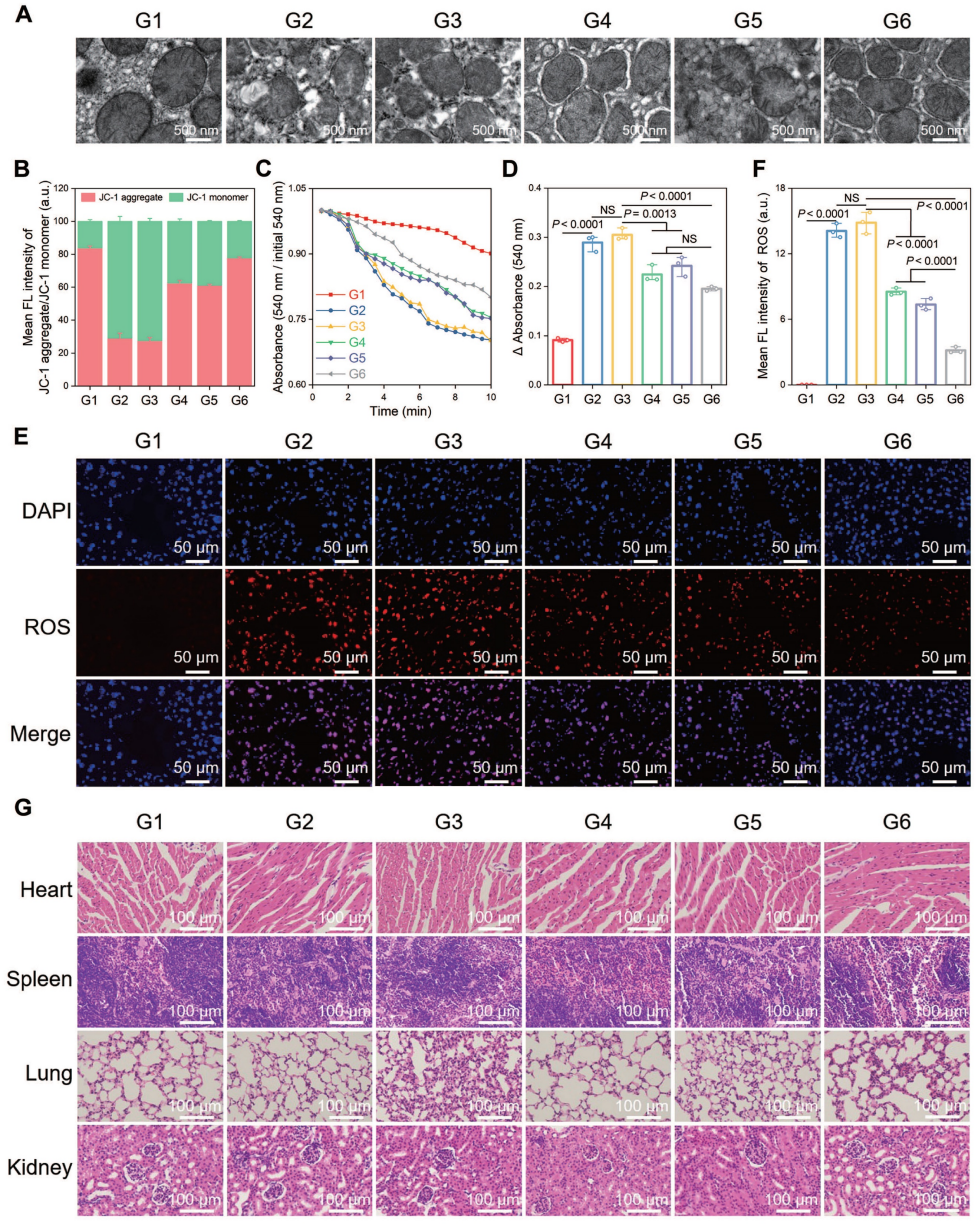
** Protective effects against mitochondrial dysfunction and biosafety of CsA/miRi@Exos^CD47-HuR^ *in vivo*.** (**A**) Morphological analysis of mouse liver mitochondria by electron microscopy. Scale bar: 500 nm. (**B**) Evaluation of Δψm by JC-1 dye in purified liver mitochondria. (**C**) Detection of mPTP opening with an absorbance assay at 540 nm, with measurements repeated every 30 sec for 20 times. (**D**) Changes in absorbance values were recorded within 10 min to indirectly evaluate the extent of mPTP opening. (**E**) ROS level detected by DCFH-DA probe in liver ischemia tissues. Scale bar: 50 μm. (**F**) Quantitative analysis of DCFH-DA fluorescence intensity. (**G**) H&E staining of major organs (heart, spleen, lung, and kidneys, respectively) to analyze the biosafety of Exos. Scale bar: 100 μm. Groups were designed as: sham (G1); BALB/c mice with HIRI (G2); BALB/c mice pretreated with Exos^CD47-HuR^ before HIRI (G3); BALB/c mice pretreated with CsA@Exos^CD47-HuR^ before HIRI (G4); BALB/c mice pretreated with miRi@Exos^CD47-HuR^ before HIRI (G5); BALB/c mice pretreated with CsA/miRi@Exos^CD47-HuR^ before HIRI (G6). miRi refers to mmu-miR-7057-3p inhibitor. Data are expressed as mean ± S.D. (n = 3), ***P < 0.001, **P < 0.01, *P < 0.05 NS means no difference (non-repeated ANOVA followed by Tukey's test).

A terminal deoxynucleotidyl transferase (TdT) dUTP nick end labeling (TUNEL) assay was performed to observe cell apoptosis, and a significant increase in the number of TUNEL-positive cells was observed after HIRI, which was notably decreased in the CsA/miRi@Exos^CD47-HuR^ group (Figure 5E and F). Western blot analysis of apoptosis-related proteins presented the same results (Figure 5G and H). Additionally, western blot and qRT-PCR analysis of residual liver tissue showed the upregulation in TFAM protein and mRNA levels after treatment with CsA/miRi@Exos^CD47-HuR^, revealing that CsA/miRi@Exos^CD47-HuR^ possessed anti-apoptotic potential which might depend on the upregulation of TFAM (Figure 5G-I). Moreover, the impact of CsA/miRi@Exos^CD47-HuR^ on mitigating mitochondrial damage was examined. First, electron microscopic showed that HIRI damaged mitochondrial morphology in the liver, which could be ameliorated by CsA/miRi@Exos^CD47-HuR^ (Figure 6A). To further examine the alterations in mitochondrial function, Δψm and mPTP opening in mouse hepatocytes were assessed using a JC-1 assay and a colorimetric approach, respectively (Figure 6B-D), and ROS levels were evaluated by the DCFH-DA assay (Figure 6E and F). There was a significant decrease in Δψm, the extent of mPTP opening, and an increase in ROS levels in the HIRI groups compared to the sham group. In contrast, treatment with drug-loaded Exos^CD47-HuR^ increased Δψm, inhibited mPTP opening, and reduced ROS production, suggesting their capacity to effectively protect mitochondrial function during HIRI *in vivo*. Finally, major organs (heart, spleen, lung, and kidneys) in the sham and other treatment groups were harvested to evaluate the potential side effects of Exos with or without cargos by H&E staining (Figure 6G). Mice treated with CsA/miRi@Exos^CD47-HuR^ exhibited no discernible organ impairment or apparent inflammation or necrosis, indicating that CsA/miRi@Exos^CD47-HuR^ possessed good biosafety for the treatment of HIRI. Taken together, our findings demonstrated that CsA/miRi@Exos^CD47-HuR^ could evade immune surveillance by the “camouflage cloak” of CD47. Once endocytosed by hepatocytes, Exos could release the encapsulated cargos, which protected mitochondria and alleviated liver damage caused by HIRI.

## Discussion

We successfully reprogrammed a potent and adaptable drug delivery system that could bolster the loading capability of dual therapeutic ingredients and evade immune surveillance to achieve targeted transport, which stabilized mitochondrial function and ultimately prevented the progression of HIRI. After engineering Exos to be “invisible” to macrophages, we demonstrated the feasibility of CsA/miRi@Exos^CD47-HuR^ to protect mitochondrial function in LO2 cell models of OGD/R and mouse models of partial (70%) warm HIRI. This study is the first attempt to reprogram Exos for the precise dispatch of polypeptides and RNA and offers therapeutic prospects in the clinical treatment of HIRI.

As a crucial cause of liver damage during surgical procedures 2, HIRI is closely associated with the prognosis of patients undergoing hepatic resection, liver transplantation, or trauma procedures. Multiple strategies have been explored to lessen the harmful consequences of HIRI, such as ischemic preconditioning, which involves subjecting an organ to a brief period of ischemia before a more sustained ischemia/reperfusion period 29. Ischemic preconditioning has been demonstrated to provide benefits and alleviate HIRI in rodent models and patients undergoing liver transplantation or major resection of the liver 30. However, the absence of a standardized protocol for the optimal duration and number of ischemic preconditioning cycles has hindered the widespread promotion of this technique 31. In addition, hypothermic machine perfusion, which is another preconditioning method, is limited by the requirement for specialized equipment and expertise 32. Various pharmacological interventions, such as antioxidants, adenosine agonists, pentoxifylline, protease inhibitors, and prostaglandins, have been studied to counteract the detrimental impact of HIRI 33. Although the aforementioned preventive measures can effectively reduce liver damage, challenges still exist in determining specific interventions and minimizing side effects 33. While reviewing most methods, the primary priority was to preserve ATP and reduce oxidative stress, particularly by scavenging ROS. As energy factories and major sources of ROS production in cells, mitochondria are targets for ameliorating liver dysfunction. In the initial stage of HIRI, mitochondrial dysfunction is characterized by the opening of mPTP and subsequent CytC release, which leads to consequent hepatocyte death 34. In addition, a decrease in TFAM, a nuclear-encoded protein that facilitates the maintenance of mtDNA integrity and prevents mutation accumulation, is a characteristic of the early phase of HIRI and further aggravates respiratory chain dysfunction, leading to mitochondrial deficiencies 10, 35, 36. Under these circumstances, suppressing mPTP opening and upregulating TFAM expression in dysfunctional mitochondria is crucial in the initial stage to prevent subsequent irreparable damage. As a polypeptide that competitively inhibits CypD, CsA can suppress the opening of mPTP. On the other hand, as a noteworthy therapeutic molecule 37, 38, miRi can upregulate TFAM, which was demonstrated in our study. However, systemic administration of CsA inevitably causes widespread toxicity due to its poor solubility and nonselective distribution *in vivo* 39. Recent delivery systems for miRi, such as viral vectors, poly(lactide-co-glycolide) particles, and neutral lipid emulsions, have limitations about loading capacity, targeting efficiency to cells, and toxicity 40. In general, searching for a golden curecourier to safely and effectively deliver CsA and miRi to hepatocytes has considerable potential for the treatment of HIRI 41.

As intercellular communicators, Exos exhibit natural drug delivery properties 42. However, the presence of macrophages which are responsible for engulfing foreign particles, especially Kupffer cells in the liver, continues to be a major challenge in the delivery of Exos to hepatocytes 43. Thus, strategies to generate Exos that are “invisible” to immune surveillance, especially in the liver are urgently needed. Based on the mechanisms by which tumor cells evade macrophage phagocytosis as a result of their high expression of CD47 44, we engineered Exos to transmit a “don't eat me” signal to achieve targeted transport to hepatocytes. As expected, our study demonstrated that the targeted delivery of Exos^CD47-HuR^ by evading macrophage phagocytosis was superior to that of Exos^LO2^ both *in vitro* and *in vivo*. After achieving targeted transport, the major challenge was to enhance the loading of therapeutic agents in Exos^CD47-HuR^. About the loading of CsA, Exos^CD47-HuR^ (LE = 15.77%) had a higher loading capability than the lipoprotein nanocarrier (LE = 7.30%) 45. To load exogenous miRi into Exos, common methods include electroporation, sonication, and the use of Lipofectamine, but their loading capability is comparatively low 46. To “seat more sites” for miRi in Exos, we borrowed the function of HuR, which can increase the efficiency of encapsulating miRi by interacting with the AREs of the target RNA 47. Thus, we fused HuR with the C-terminus of CD47 to construct the novel carrier Exos^CD47-HuR^, which had a high loading capability for miRi compared with Exos^LO2^ (1.91% vs 0.04%), as validated in our study. After being loaded with dual cargos, there was an increase in the internalization of CsA/miRi@Exos^CD47-HuR^ by hepatocytes, and CsA and miRi inside Exos^CD47-HuR^ were released into the cytoplasm 48. As anticipated, CsA/miRi@Exos^CD47-HuR^ could significantly protect mitochondrial function by restricting mPTP opening and upregulating of TFAM, which collaboratively halted the progression of hepatocyte death.

In conclusion, we have established a novel curecourier to deliver CsA and miRi to hepatocytes to alleviate HIRI. By evading immune surveillance, our reprogrammed Exos can maintain mitochondrial homeostasis during oxygen supply/demand imbalance. By focusing on the protection of mitochondria, this versatile agent can prevent hepatocyte death. Therefore, our results provide a novel combination strategy of drug and transcriptional manipulation to treat HIRI in the clinic. In light of this, the tactic of reprogrammed curecourier to precisely deliver cargo lays the initial foundation for the treatment of mitochondrial damage-related diseases.

## Experimental Section

### Materials

LO2 cells (human liver cells) were derived in our laboratory. NCTC-1469 (mouse liver cells) and human THP-1 monocytes were purchased from Procell Life Science & Technology Company (Hubei, China). Dulbecco's modified Eagle's medium (DMEM), Roswell Park Memorial Institute-1640 (RPMI 1640), and Opti-MEM were supplied by Gibco Life Technologies (Grand Island, USA). Fetal bovine serum (FBS) and Lipofectamine^TM^ 2000 were purchased from VivaCell (Shanghai, China) and Invitrogen (Carlsbad CA, USA), respectively. Horse serums were purchased from Procell (Wuhan, China). 0.25% Trypsin-EDTA solution, cell counting kit-8 (CCK-8, C0005, TargetMol, USA), penicillin-streptomycin, phenylmethanesulfonyl fluoride (PMSF), Cell Lysis Buffer for Radio Immunoprecipitation Assay (RIPA) and 5,5',6,6'-tetrachloro-1,1',3,3'-tetraethylbenzimidazolylcarbocyanine iodide (JC-1) were obtained from Beyotime Institute of Biotechnology (Shanghai, China). Exo-specific lysis buffer was purchased from Umibio Science & Technology Company (Shanghai, China). Cyclosporine A (CsA) was purchased from MCE company (New Jersey, USA). Phorbol-12-myristate-13-acetate (PMA) was supplied by Sigma-Aldrich (St Louis, USA). All other chemicals used in this work were of analytical grade without further purification and supplied by Chuandong Chemical Co., Ltd (Chongqing, China) unless specified. Deionized (DI) water (Millipore, USA, 18.2 MΩ) was used for all experiments.

### Cell culture

LO2 cells transfected with lentivirus containing CD47-HuR fusion gene was designated as LO2^CD47-HuR^ cells. Both LO2 and LO2^CD47-HuR^ cells were cultured in DMEM supplemented with 10% Fetal bovine serum (FBS) and 1% penicillin-streptomycin. NCTC-1469 cells were cultured in DMEM supplemented with 10% horse serum and 1% penicillin-streptomycin. THP-1 cells were grown in RPMI-1640 medium containing 10% FBS and 0.05 mM 2-mercaptoethanol. All cells were cultured at 37 °C and 5% CO_2_ atmosphere.

### Isolation and purification of exosomes (Exos)

After cells were cultured in serum-free cell culture medium for 48 h, a modified protocol to purify Exos was carried out as previously reported 49. Initially, cell medium containing Exos was collected by centrifugation at 300 g for 10 min and 2,000 g for 20 min to remove cells and then followed by centrifugation at 10,000 g for 30 min to eliminate dead cells and cell debris. Afterward, the resulting clear supernatant was centrifuged for 70 min at 100,000 g using a Sorvall WX 100 + (Thermo Fisher Scientific, Japan) to isolate Exos. After removing the supernatant, Exos pellets were resuspended in 1 mL PBS and mixed with an additional 25 mL cold PBS. Then, Exos pellets were subjected to ultracentrifugation at 100,000 g again for 70 min to remove residual media components and filter the supernatant through a 0.22 μm filter. The purified Exos were finally resuspended in 100 μL PBS and immediately stored at - 80 ℃. Exos derived from LO2 cells were utilized in all *in vivo* and *in vitro* experiments.

### Loading CsA and microRNA inhibitor (miRi) into Exos

Initially, 100 μg Exos, 100 μg miRi, and 100 μg CsA were mixed in 400 μL cold electroporation buffer (21% Opti-MEM reduced serum medium, 1.15 mM potassium phosphate, and 25 mM potassium chloride). The mixture mentioned above was transferred into an ice-cold cuvette (0.4 mm) and then subjected to electroporation using a Gene Pulser Xcell (BioRad, Hercules, CA) at 400 mV and 125 μF capacitance (pulse/10 ms). To remove unbound cargo, the mixture was washed twice with cold PBS via ultracentrifugation at 110,000 g for 70 min at 4 ℃ and subsequently resuspended in PBS. Exos loaded with CsA and miRi were called CsA/miRi@Exos^CD47-HuR^.

### Characterization of Exos

The size, morphology, and structure of Exos were investigated by a transmission electron microscope (TEM, JEM-1200EX, Japan). Besides, solutions of Exos were diluted to an appropriate concentration (~10^6^ particles/mL). Size distribution and zeta potential (ξ) of Exos were analyzed using a nanoparticle tracking analysis (NTA) instrument (ZetaView PMX110, Particle Metrix, Germany).

### Loading efficiency (LE) of CsA and miRi

High-performance liquid chromatography (HPLC) was applied to confirm the LE of CsA. A solution containing 100 μg CsA was prepared in 8 mL solvent, which was used as the initial concentration for loading. The peak area of CsA was measured by an HPLC instrument (Waters e2695, USA). In detail, the test samples were dissolved in a 1:1 acetonitrile-water mixture and then injected into an HPLC system separately alongside control solutions. The chromatographic conditions included a Waters 2489 UV/Vis Detector and a SunFire C18 Sum (4.6 × 150 mm Column). Analysis was conducted at a wavelength of 210 nm with the column temperature set to 35 °C. The concentration of CsA (C62H111N11O12) in the solutions was determined by calculating the ratio of peak areas between the control and test samples. The LE was calculated by the formula of (W_CsA_-W_free CsA_)/W_Exos_ × 100%, where W_CsA_ was the total weight of CsA, W_free CsA_ was the weight of unbounded CsA, and W_Exos_ was the weight of Exos. The LE of miRi was assessed using a spectrophotometer. The miRi used in this study was labeled with Cy5, with excitation/emission wavelengths of 650 nm/670 nm, respectively. After electroporation, Exos were incubated at 37 °C for 1 h and ultracentrifuged (110,000 g, 4 °C, 70 min) to remove unbound CsA and miRi. An equal amount of Cy5-miRi dissolved in 2 mL DEPC water was referred to as a positive control. The LE was calculated by: (W_miRi_-W_free miRi_)/W_Exos_ × 100%, where W_miRi_ was the weight of total miRi, W_free miRi_ was the weight of unbounded miRi, and W_Exos_ was the weight of Exos.

### Cell uptake of Exos

Exos were labeled with PKH26 dye (red). An appropriate amount of Exos was taken to determine their protein concentrations using a BCA assay. The “PKH26 linker (for red fluorescent cell labeling)” storage solution was diluted 10 times with “Diluent C” to prepare a dye working solution with a concentration of 100 μM. The operation was carried out in the dark and the working solution was prepared according to the experimental usage. 100 μg Exos was added to 50 μL working solution. After adding the dye working solution, the centrifuge tube was tightly capped and mixed for 1 min using a vortex mixer, followed by a 10 min incubation. Next, 10 mL PBS was added to the Exo-dye complex and mixed well. The Exos were extracted again using ultracentrifuge to remove excess dye. The precipitate, which was the stained Exos, was resuspended in 200 μL PBS. Cells were cultured in a 24-well plate and allowed to adhere for a minimum of 12 h. Then, PKH26 labeled Exos were added to the cells at a concentration of 50 μg/mL. After incubation for 2 or 4 h at 37 °C, the cells were washed with PBS buffer. To visualize the cellular uptake of Exos, cell membranes were stained with 3,3'-dioctadecyloxacarbocyanine perchlorate (DIO, green) and nuclei were stained with 4',6-diamidino-2-phenylindole (DAPI, blue). Confocal laser scanning microscopy (CLSM, Leica TCS SP8, Mannheim, Germany) was used for imaging.

### *In vitro* cargo release assay

The quantities of CsA and miRi were calculated using a standard curve. For the CsA release kinetics, CsA/miRi@Exos^CD47-HuR^ (50 μgs of total protein) were suspended in 0.1 mL of PBS, loaded into Slide-A-Lyzer MINl dialysis units (molecular mass cutoff 10 kDa, Pierce), and dialyzed against 1 mL of PBS at 37 °C with continuous agitation at 250 rpm. The supernatant was harvested at 0, 2, 4, 8, 12, 24, 36, and 72 h. CsA content in samples was quantified using UV-visible spectroscopy at a wavelength of 210 nm, while the miRi content was determined using a spectrophotometer with excitation and emission wavelengths set at 650 nm and 670 nm, respectively.

### OGD/R model

To establish the oxygen-glucose deprivation/reoxygenation (OGD/R) model, cellular hypoxia was ensured by exposure to the air condition of 1% O_2_, 5% CO_2_, and 94% N_2_. First, the hypoxia was induced for 2, 4, 6, 8, and 10 h, respectively, and the fixed reoxygenation time was set for 24 h. Then, the hypoxia time was fixed at 6 h, and reoxygenation was conducted for 3, 6, 12, and 24 h, respectively. Ultimately, the optimal time points for OGD/R were ascertained by the expression of TFAM and apoptosis-related protein.

### Evaluation of the capability of escaping from the immune surveillance *in vitro*

THP-1 cells were seeded in the 24-well plates and incubated with 100 ng/mL of PMA for 48 h before the phagocytosis assay. Exos^LO2^ and Exos^CD47-HuR^ labeled by PKH26 dye were co-cultured with differentiated THP-1 cells for 2 h and 4 h, respectively. Subsequently, the cells were stained with DIO and DAPI and observed using CLSM.

### *In vivo* biodistribution

The biodistribution of Exos was investigated *in vivo* in this study. Exos were labeled with 1,1'-dioctadecyl-3,3,3',3'-tetramethylindotricarbocyanine iodide (DIR) dye (AAT Bioquest, USA) at a final concentration of 1 μM, with 200 μg of Exos in a volume of 200 μL, and were administered intravenously via the tail vein. Fluorescence signals were captured using a fluorescent imaging system (NightOWL II LB983, Berthold, Bad Wildbad, Germany) with excitation and emission wavelengths of 750/780 nm at 0.5 h, 3 h, 6 h, 12 h, 24 h, 48 h, and 72 h post-injection. Major organs, including the liver, lung, spleen, heart, and kidney, were harvested 24 h post-injection when the mice were humanely euthanized and were imaged using the same equipment. The mean fluorescence intensities were analyzed using Indigo software.

### Establishment of BALB/c mouse hepatic ischemia/reperfusion injury (HIRI) model

Male BALB/c mice aged 6~8 weeks were procured from the Animal Center of Chongqing Medical University. The animal experiments were performed by the Guide for the Care and Use of Laboratory Animals and approved by the Animal Ethics Committee at Chongqing Medical University (Chongqing, China). Surgical induction of HIRI or sham surgery was conducted as previously reported 50. Briefly, mice were anesthetized via intraperitoneal injection of sodium pentobarbital (60 mg/kg) and placed supine on a temperature-controlled surgery table set at 37 °C to maintain the mice's body temperature at 33 °C. A midline laparotomy was performed, and an atraumatic clip was employed to block clamp blood supply to the left lateral and median lobes of the liver. Following 60 min of partial hepatic ischemia, the clip was removed to initiate hepatic reperfusion. Sham control underwent identical procedures without vascular occlusion. The mice were kept hydrated with warm saline throughout the procedure. The mice were euthanized at several fixed reperfusion time points (3 h, 6 h, 12 h, 24 h, 36 h, 48 h, 72 h) after reperfusion to collect blood and the ischemic liver tissue for further analysis. Each animal experiment consisted of three replicates within each group of animals.

### TUNEL

Saline or different kinds of drug-loaded Exos^CD47-HuR^ were intravenously injected to mice via the tail vein 24 h before HIRI. After HIRI, the mice were euthanized and their liver tissues were carefully collected. The tissue sections were subjected to a dewaxing process using a series of solutions including xylene Ⅰ, xyleneⅡ, ethanol Ⅰ, ethanol Ⅱ, 85% ethanol, 75% ethanol, and distilled water wash. To ensure proper fixation, a histological pen was used to circle the tissue, and 100 μL 1×proteinase K working solution was added for 20 min at 37 °C. Following fixation, the slides were washed 3 times with PBS for 5 min each. Subsequently, 100 μL TDT equilibration buffer was added to each sample and incubated at 37 °C for 30 min. After washing with PBS, 50 μL labeling working solution was added to each sample and incubated at 37 °C for 1 h in a humid chamber. The sections were then stained with DAPI for 10 min at room temperature and washed again with PBS. The sections were slightly dried and sealed with an anti-fluorescence quenching sealing agent. Finally, the slides were examined under a fluorescence microscope and images were acquired.

### Mitochondrial morphology by TEM

Liver tissues were carefully dissected into 0.5~1.0mm^3^ blocks using a precise cutting tool to minimize tissue distortion. The tissue samples were rapidly immersed in 3% precooled glutaraldehyde fixative within a time frame of no more than 1 min, to optimize the preservation of the tissue's native cellular structure. Firstly, it was washed 4 times with PBS for 15 min each to remove any extraneous material. Then, it was fixed with 1% osmium tetroxide for 2 h to preserve its ultrastructure. Afterward, it was washed again with PBS 4 times for 15 min each to remove any excess fixative. The sample was then dehydrated with a series of acetone gradients (50%, 70%, 90%, 100%) for 15 min each at 4 °C to remove water and prepare it for embedding. It was infiltrated with embedding medium and cured in a 60 °C oven, with the curing process consisting of 12 h at 35 °C, 12 h at 45 °C, and 2 days at 60 °C to ensure the sample was firmly embedded in the medium. The sample was sliced into ultra-thin sections of 60~80 nm, stained with lead citrate and uranyl acetate, washed with distilled water, and air-dried. Finally, the ultrastructure of the sample was observed under TEM, and images were captured.

### Statistical analysis

OriginPro 2023 (version 10.0.0.154) was used to draw histograms and line plots. Data are shown as mean ± S.D. of triplicates unless otherwise indicated. Statistical analysis was performed using a two-tailed Student's t-test or one-way ANOVA with post hoc tests, as appropriate. No animals were excluded from the analysis. *P* value less than 0.05 was designated statistically significant. Fluorescence Images were quantitatively analyzed using ImageJ (version 1.52). Statistical analysis was performed with GraphPad Prism software (version 9.0.0).

### Author contributions

**Shanshan Liu:** Conceptualization, Methodology, Investigation, Data Curation, Writing - Original draft preparation. **Xinyu Xiao:** Conceptualization, Methodology, Writing - Original draft preparation. **La Zhang:** Conceptualization, Writing - Original draft preparation. **Jianwei Wang:** Validation, Investigation. **Wei Zhao:** Methodology. **Haichuan Liu:** Methodology. **Mengxia Xu:** Visualization. **Rui Liao:** Visualization. **Jiao Guo:** Methodology. **Zhi Li:** Supervision. **Baoyong Zhou:** Methodology, Supervision. **Chengyou Du:** Writing - Review & Editing, Supervision. **Qiling Peng:** Funding acquisition, Writing - Review & Editing, Supervision. **Ning Jiang:** Funding acquisition, Writing - Review & Editing, Supervision.

### Data availability

The data supporting the findings of this study are available within the article and its Supplementary Information. All other data are available from the corresponding author upon request. Source data are provided in this paper.

## Supplementary Material

Supplementary methods, figures and tables.
