# Supplementary material for: Reprogramming Exosomes to Escape from Immune Surveillance for Mitochondrial Protection in Hepatic Ischemia-Reperfusion Injury
Source: Theranostics. 2024 Jan 1;14(1):116–32. doi: 10.7150/thno.88061 (PMC10750206; doi:10.7150/thno.88061)
Supplement: Supplementary file 1 — Supplementary methods, figures and tables. [file thnov14p0116s1.pdf]

Reprogramming Exosomes to Escape from Immune Surveillance for Mitochondrial Protection in Hepatic Ischemia-Reperfusion Injury

Shanshan Liu[1,8]† Xinyu Xiao[1]†, La Zhang[2]†, Jianwei Wang[1]†, Wei Zhao[1], Haichuan Liu[2], Rui Liao[2], Zhi Li[3], Mengxia Xu[3], Jiao Guo[1], Baoyong Zhou[2]*, Chengyou Du[2]*, Qiling Peng[1,4]* & Ning Jiang[5,6,7]*

[1] School of Basic Medical Science, Chongqing Medical University, Chongqing 400016, P. R. China. [2] Department of Hepatobiliary Surgery, the First Affiliated Hospital of Chongqing Medical University, Chongqing 400016, P. R. China. [3] Traditional Chinese Medicine Hospital of Bijie City, Guizhou province, 551700, People's Republic of China. [4] Bijie Municipal Health Bureau, Guizhou province, 551700, People's Republic of China. [5] Department of Pathology, Chongqing Medical University, Chongqing 400016, P. R. China. [6] Molecular Medicine Diagnostic and Testing Center, Chongqing Medical University, Chongqing 400016, P. R. China. [7] Department of Pathology, the First Affiliated Hospital of Chongqing Medical University, Chongqing 400016, P. R. China. [8] Department of Plastic and Maxillofacial Surgery, The Second Affiliated Hospital of Chongqing Medical University, Chongqing 400016, P. R. China.

*Corresponding author. E-mail: sdlytczhzh@163.com (Baoyong Zhou); duchengyou@126.com (Chengyou Du); pqlpzy@cqmu.edu.cn (Qiling Peng); jiangning@cqmu.edu.cn (Ning Jiang)

† These authors contributed equally to this work.

Supplementary Methods

Lentivirus-mediated transfection of CD47-HuR fusion gene into LO2 cells

Lentiviral vectors GV367 for stable expression of CD47-HuR fusion gene were successfully synthesized by GeneChem Incorporation (Shanghai, China). They carry fragments to express green fluorescent protein (GFP) and puromycin resistance protein (PAC), which are shown in Figure S8. Then, vectors incorporated with CD47-HuR fusion protein were fabricated. Specific primers used for validating the fusion gene by GeneChem were listed in Table S1. LO2 cells were seeded in 24-well plates (5×10^4 cells/well) for lentiviral transfection following the previous methods [1], and were named $LO2^{CD47\text{-}HuR}$ cells.

Transient transfection of microRNA (miR) modulators into LO2 and NCTC-1469 cell

Hsa-miR-590-3p and mmu-miR-7057-3p mimics, inhibitors, and negative controls (NC) were synthesized by Tsingke Biotechnology (Beijing, China). Cells were seeded in 6-well plates at a density of 2.5×10^5 cells/well and incubated for 24 h before transfection. Transfection mixes A and B were prepared separately for each well. Mix A contained 250 µL Opti-MEM and 5 µL mimics/inhibitors, while mix B contained 250 µL Opti-MEM and 5 µL Lipofectamine™ 2000. Both mixtures were vortexed together for 10 sec and incubated for 5 min. The culture medium was removed from cells and the cells were rinsed with PBS. Then, 1 mL medium was added to the cells followed by an additional 500 µL transfection mixture (A+B) to the well. After transfection, cells should then be incubated in a fresh medium for another 48 h. The sequences of miR-590-3p and miR-7057-3p mimics/inhibitors are shown in Table S2.

Extraction of protein from cells, liver tissue, and Exos

To prepare the cellular lysate for western blot, 0.5 mL RIPA containing 1% PMSF was added to the cells after being rinsed with PBS thrice. Cells were then lysed for 30 min at 4 °C. Besides, dissected liver tissues were precisely weighed and placed into a Precellys tissue grinding tube. Subsequently, the tissues were lysed with cell lysis buffer (20 µL/mg) and thoroughly ground until most of the tissues were dissolved. To extract proteins from Exos, the sample was added to Exo-specific lysis buffer at a volume ratio of 1:1, and the resulting mixture was incubated on ice for 10 min for efficient lysis. Afterward, these supernatants were collected by centrifugation at a speed of 12,000 rpm for 15 min. The extracted proteins were then subjected to protein quantification and western blot.

Bicinchoninic acid (BCA) protein assay and western blot

The BCA protein assay kit was used to determine the protein concentration according to the manufacturer's instructions., and the expression levels of interest proteins were examined by western blot. Briefly, equal amounts (30 µg) of sample proteins were loaded onto a well of polyacrylamide gel electrophoresis (PAGE) and electrophoresis was run at 80 V for 30 min, followed by 110 V for 50 min. Afterward, the proteins were transferred to polyvinylidene difluoride (PVDF, Millipore, USA) membranes at a current of 250 mA for 60~120 min. Then, PVDF membranes were blocked with 5% skim milk powder for 60 min at room temperature, and rinsed with 1× Tris-buffered saline containing 1% Tween 20 (TBST) buffer. After that, the PVDF membranes were incubated with primary antibodies at 4 °C overnight and subsequently horseradish peroxidase (HRP)-conjugated goat anti-rabbit or goat anti-mouse secondary antibody in 1× TBST buffer at room temperature with shaking for 60 min. β-actin was referred to as control. Finally, the expression levels of proteins were monitored by an enhanced chemiluminescent (ECL) reagent (Life-iLab, China) and visualized with an ECL detection system (BIO-OI, China). The detailed information of primary and secondary antibodies used in this study and their concentrations are shown in Table S3.

RNA extraction

Enzyme-free EP tubes, reagents, and consumables were exposed to UV irradiation for sterilization. Cells were gently washed thrice for RNA extraction. Then, appropriate amounts of Trizol (Takara, Japan) were added (1 mL/well). After shaking, the cell suspension was transferred by pipette into an enzyme-free 1.5 mL EP tube, added with 200 μL chloroform, and centrifuged at 12,000 rpm for 15 min at 4 ℃. After that, the colorless aqueous phase layer was transferred into a new EP tube. 700 μL isopropanol was added, and the mixture was centrifuged at 12,000 rpm for 10 min at 4 ℃. Next, the supernatant was discarded. Subsequently, the concentration of RNA was measured using a Nanodrop 2000 spectrophotometer (Thermo Fisher Scientific, USA). Finally, the RNA samples were stored at − 80 ℃ for further use.

Quantitative Real-Time PCR (qRT-PCR)

Firstly, extracted RNAs (1 μg) were placed into sample tubes. Then, 1 μL 5× gDNA Eraser Buffer (No.2) and gDNA Eraser (No.1) were added to each sample tube. The remaining volume was supplemented with diethylpyrocarbonate (DEPC) water, and the mixture was allowed to react at room temperature for 15 min. To perform RNA reverse transcription, the reaction mixture was added with the following components: 1 μL PrimerScript RT Enzyme Mix I (No.3), 4 μL 5× PrimerScript Buffer 2 (No.4), 1 μL RT Primer Mix (No.5), and 4 μL DEPC water (No.6). The reaction was performed at 37 °C for 15 min and terminated by heating at 85 °C for 5 s. The resulting cDNAs were diluted 5~10 times and stored at – 20 °C for further use. For fluorescent quantitation, 12.5 μL TB Green Premix Ex TaqII and 10.3 μL DEPC water were added to mixtures. The resulting mixtures were then added to an 8-tube strip with each tube containing 2 μL cDNA from different experimental groups. The PCR amplification was performed using a fluorescence quantitative PCR instrument. The process started with a pre-denaturation step at 95 ℃ for 30 sec, followed by 40 cycles of denaturation at 95 ℃ for 5 sec, annealing at 60 ℃ for 30 sec. The data analysis was performed using the $2^{-\triangle\triangle}$CT formula. The primer sequences for SybrGreen were designed by Sangon Biotech (Shanghai, China) and were listed in Table S4.

Cell viability assay

For cytocompatibility testing, LO2 cells were seeded in 96-well plates, 24-well plates, or 6-well plates. 24 h after cell adhesion, the fresh cell culture medium containing $Exo^{CD47-HuR}$, $CsA@Exo^{CD47-HuR}$, $miRi@Exo^{CD47-HuR}$, or $CsA/miRi@Exo^{CD47-HuR}$ were added for incubation (24 h). After OGD/R, the cells were stained using the LIVE/DEAD Fixable Dead Cell Stain kit (Beyotime, China) to differentiate between live and dead cells. The cell viability was assessed three times using a microplate reader (Thermo Fisher, USA) by measuring the absorbance at 450 nm with a cell counting kit-8 assay. Besides, the Annexin V-FITC/PI (AV/PI) apoptosis assay kit (Beyotime, China) was used to distinguish apoptotic cells. Then a flow cytometer (CytoFLEX, BeckmanCoulter, USA) was used to measure the apoptosis rates of LO2 cells. Flowjo software (version 10.8.1) was used to analyze the results.

Observation of mitochondrial morphology by CLSM

To observe the mitochondrial morphology, LO2 cells were incubated with different groups of Exos for 24 h on a 24-well glass slide and then suffered from OGD/R. After that, MitoTracker was used to stain active mitochondria for 30 min at 37 ℃, followed by rinsing 3 times by PBS and fixed with 4% paraformaldehyde in Hanks' balanced salt solution (HBSS) for 15 min. The cell nuclei were stained blue using DAPI for 30 min. After washing 3 times in PBS, CLSM equipped with a 63×/1.4 NA oil objectives and laser/filter sets was used to observe DAPI (blue) and MitoTracker (red).

Characterization of the mitochondrial membrane potential (ΔΨm)

ΔΨm was assessed in live cells using JC-1. LO2 cells were seeded in 6-well plates or 24-well plates and treated with different groups of Exos for 24 h. After washing with PBS, each well was mixed with 0.1% JC-1 and incubated for 20 min at 37 °C. 2 mL cell culture medium was added to each well and washed twice with JC-1 staining buffer. Images were scanned by CLSM, and the CytoFLEX flow cytometer was also used to determine ΔΨm. The ratio of JC-1 aggregate/JC-1 monomer fluorescence intensity was carried out to measure ΔΨm.

Assessment of mitochondrial permeability transition pore (mPTP) opening

Calcein-AM/$CoCl_2$ assay (Beyotime, China) was performed to evaluate the opening condition of the mPTP as described previously [2]. LO2 cells were seeded in 24-well plates, incubated with different groups of Exos for 24 h, and washed with PBS twice. Each well was incubated with Calcein-AM (2 μM) for 30 min at 37 °C. After washing in PBS twice, the cells were exposed to $CoCl_2$ (2 mM) for 15 min to detect the distribution of cobalt inside mitochondria. The degree of mPTP opening was reflected by green (Calcein-AM) fluorescence assessed by CLSM and the CytoFLEX flow cytometer.

Measurement of intracellular reactive oxygen species (ROS) and mitochondrial ROS

After LO2 cells were treated as described above, the ROS assay kit (Beyotime, China) was used to assess the intracellular ROS levels in accordance with the manufacturer's instructions. In brief, LO2 cells were stained with 2',7'-dichlorofluorescein diacetate (DCFH-DA, green) probe for 30 min in the dark and the fluorescence images were captured by CLSM. MitoSox Red (Yeasen Biotechnol., China), a probe specifically targeting mitochondria, was used to selectively detect hyperoxide produced by mitochondria as previously described [3]. LO2 cells were loaded with 5 μM MitoSox Red diluted in HBSS for 10 min at 37 °C. The fluorescence intensity was observed under CLSM.

Transaminase detection

Mouse blood samples were collected using the eye-ball bleeding method with EDTA-Na2 anticoagulant tubes. The samples were then centrifuged at 2,000 rpm for 15 min, and the plasma was tested for the activity of ALT and AST using an automated blood biochemical analyzer.

Hematoxylin & Eosin (H&E) staining

First, tissues were gently rinsed with PBS buffer or physiological saline, and then we fixed the tissues by soaking them in a 4% paraformaldehyde solution. Next, a defatting agent was applied to remove water and fat. The samples were then immersed and embedded in melted paraffin to fix and protect their morphology. Then, the embedded tissues were cut into thin slices of 5~8 μm using a microtome. H&E staining was performed as previously reported [4] and analyzed under a microscope (Nexcope NIB410, USA). The Suzuki score [5] was used to evaluate the degree of liver tissue damage after HIRI, and the details are described in Table S5.

Detection of ROS in liver tissues

First, frozen sections were thawed at room temperature and the moisture content was controlled. A circle was drawn around the tissue with a histological pen, and a self-quenching fluorescent reagent was added for 5 min, followed by rinsing with running water for 10 min. Then, the red fluorescence probe Dihydroethidium (DHE, Sigma-Aldrich, Shanghai, China) was added to the circle, and the

samples were incubated at 37°C in the dark for 30 min. The slides were washed on a shaking platform in PBS 3 times, each time for 5 min. DAPI staining solution was added and the samples were incubated at room temperature in the dark for 10 min. The slides were washed again on the shaking platform in PBS 3 times, each time for 5 min. Finally, the anti-fluorescence quenching sealing agent was added for slide sealing.

Tissue mitochondrial purification

All solutions in the kit (Beyotime, China) were thawed at room temperature, mixed thoroughly, and immediately placed on ice. After completing the animal experiments, a small piece of liver left lobe tissue weighing approximately 50~100 mg was excised and weighed in a 1.5 mL centrifuge tube. The tissue was washed once with PBS and placed in a centrifuge tube on ice. Next, 10 volumes of pre-cooled mitochondrial separation reagent A were added, and the tissue was homogenized on ice approximately 10 times. Note: If the tissue weighs 80 mg, it can be roughly estimated that the tissue volume is close to 80 μL, and thus 800 μL of mitochondrial separation reagent A should be added. The homogenate was centrifuged at 1,000 g and 4 °C for 5 min to obtain higher purity mitochondria. The supernatant was carefully transferred to another centrifuge tube and centrifuged at 3,500 g and 4 °C for 10 min. The supernatant was carefully removed, and the precipitate was the isolated mitochondria. Typically, 40 μL corresponding mitochondrial storage solution could be used to resuspend the mitochondria obtained from 100 mg tissue. The expected protein concentration of the isolated mitochondrial sample was 10~20 mg/mL. Then, the extracted mitochondria were subjected to subsequent detection within 6 h.

Detection of ΔΨm in liver tissues by JC-1

Dilute 0.9 mL JC-1 staining working solution and add 0.1 mL purified tissue mitochondria with a total protein content of 10~100 μg. Mix the samples and measure them using a spectrofluorometer FS5 (Edinburgh Instruments, UK). The green fluorescence was detected at an excitation wavelength of 490 nm and an emission wavelength of 530 nm, while the red fluorescence was measured using an excitation wavelength of 525 nm and an emission wavelength of 590 nm. The ratio (red/green) was calculated to evaluate ΔΨ. A smaller ratio indicated lower ΔΨ and therefore decreased mitochondrial function, which could be used to evaluate cellular apoptosis and mitochondrial function.

Detection of mPTP opening in liver tissue

The purified mPTP colorimetric assay kit (GENMED, USA) was used to determine the degree of mPTP opening. Firstly, the tested purified mitochondrial samples were prepared and transferred into the corresponding wells of a 96-well enzyme-linked immunosorbent assay plate. Next, GENMED buffer solution (Reagent A) was added, mixed, and left to stand for 1 min at room temperature. Then, an induction solution (Reagent B) was added and the samples were immediately placed into a spectrophotometer (at a wavelength of 540 nm), and the initial absorbance (Initial A540) was recorded. Subsequently, the absorbance ratio (A540/Initial A540) was calculated at each time point by monitoring continuously every 30 s for 10 min, which represented the actual absorbance value at the designated time divided by the absorbance value at 0 min. If the absorbance ratio decreased, it indicated an increased activity of mPTP. The membrane channel pore induction opening curve was constructed, in which the absorbance ratio (A540/Initial A540) was used as the vertical axis (Y) and the detection times were used as the horizontal axis (X).

References

1. Cadena C, Ahmad S, Xavier A, Willemsen J, Park S, Park JW, et al. Ubiquitin-Dependent and -Independent Roles of E3 Ligase RIPLET in Innate Immunity. Cell. 2019; 177: 1187-200.e16.

2. Petronilli V, Miotto G, Canton M, Colonna R, Bernardi P, Di Lisa F. Imaging the mitochondrial permeability transition pore in intact cells. Biofactors. 1998; 8: 263-72.

3. Chen B, Li H, Ou G, Ren L, Yang X, Zeng M. Curcumin attenuates MSU crystal-induced inflammation by inhibiting the degradation of IκBα and blocking mitochondrial damage. Arthritis Res Ther. 2019; 21: 193.

4. Liu B, Li F, Shi J, Yang D, Deng Y, Gong Q. Gastrodin Ameliorates Subacute Phase Cerebral Ischemia-Reperfusion Injury by Inhibiting Inflammation and Apoptosis in Rats. Mol Med Rep. 2016; 14: 4144-52.

5. Suzuki S, Toledo-Pereyra LH, Rodriguez FJ, Cejalvo D. Neutrophil infiltration as an important factor in liver ischemia and reperfusion injury. Modulating effects of FK506 and cyclosporine. Transplantation. 1993; 55: 1265-72.

Supplementary Figures

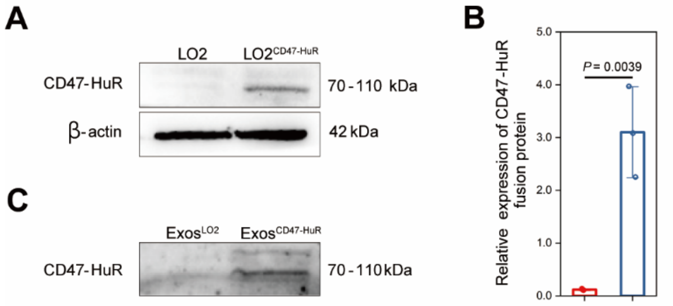

Figure S1 Human liver (LO2) cells functionalization with CD47-HuR. (**A**) Western blot showing the expression differentiation of CD47-HuR fusion protein between LO2 and LO2$^{CD47\text{-}HuR}$ cells. (**B**) Quantitative analysis of CD47-HuR protein expression. (**C**) Western blot of CD47-HuR fusion protein from Exos^{LO2} and Exos$^{CD47\text{-}HuR}$. Data are mean ± S.D. (n = 3), (Student's t-test).

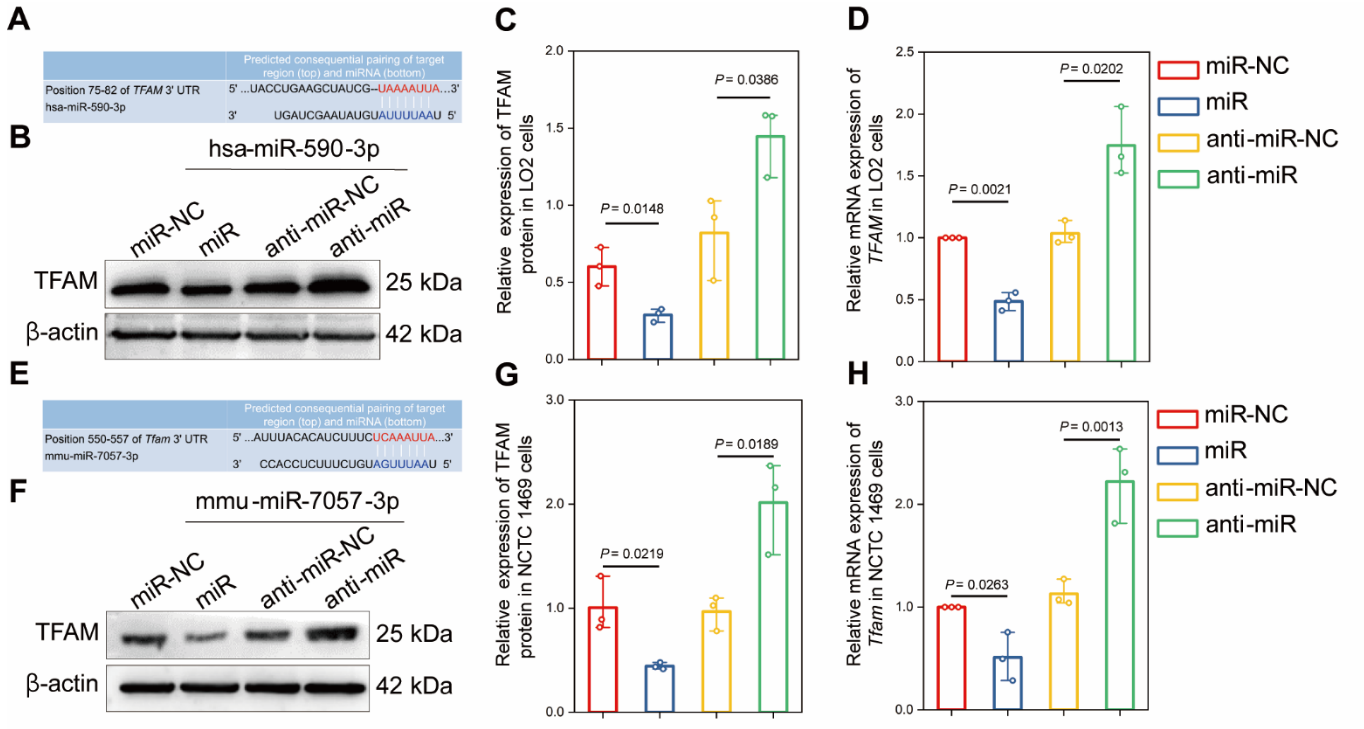

Figure S2 Identification and validation of miRNA (miR) modulators regulating TFAM. The putative seed-matching sites between hsa-miR-590-3p (**A**) or mmu-miR-7057-3p (**E**) and TFAM predicted by the TargetScan database. Protein expression of TFAM detected by western blot was significantly high after transfected with hsa-miR-590-3p inhibitor in LO2 cells (**B, C**) as well as mRNA expression of *TFAM* by qRT-PCR (**D**). Protein expression of TFAM detected by western blot was significantly high after transfected with mmu-miR-7057-3p inhibitor in NCTC-1469 cells (**F, G**) and mRNA expression of *Tfam* by qRT-PCR (**H**). miR-NC represents negative control of miR inhibitor, and anti-miR represents miR inhibitor. Data are mean ± S.D. (n = 3), (Student's t-test).

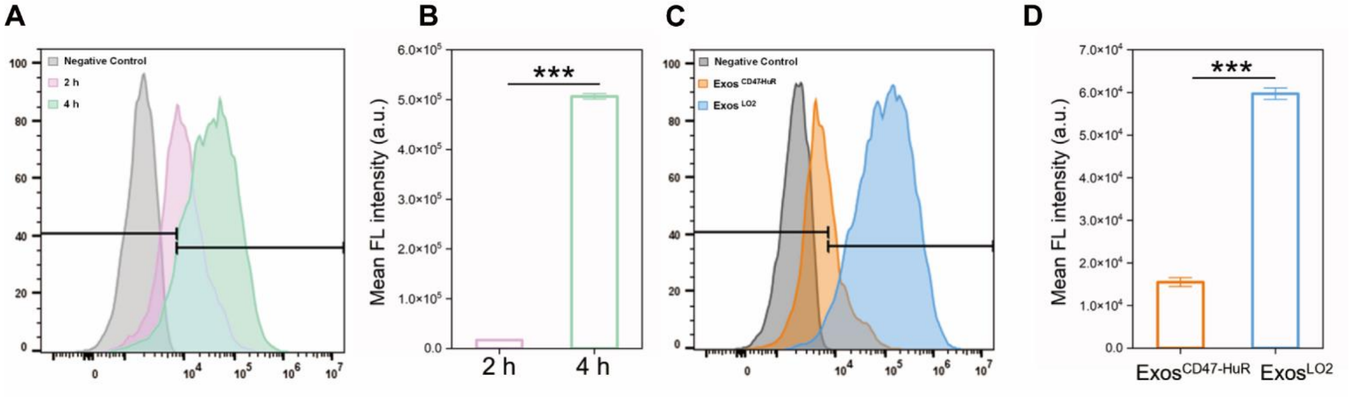

Figure S3 Flow cytometry analysis of exosome uptake. (**A**) Flow cytometry analysis of LO2 cells after co-incubation with Exos for 2 h and 4 h, respectively, and (**B**) quantitative analysis. (**C**) Flow cytometry analysis of THP-1 cells after co-incubation with $Exos^{LO2}$ and $Exos^{CD47\text{-}HuR}$ for 4 h, respectively, and (**D**) quantitative analysis. Data are expressed as mean ± S.D. ($n = 3$), ***$P < 0.001$, (Student's t-test).

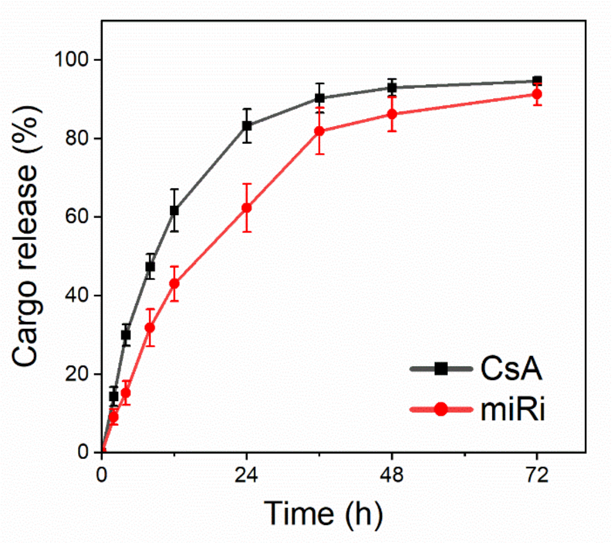

Figure S4 The accumulated release rate of CsA and miRi from Exos$^{CD47\text{-}HuR}$ at 0, 2, 4, 8, 12, 24, 36, 48, and 72h.

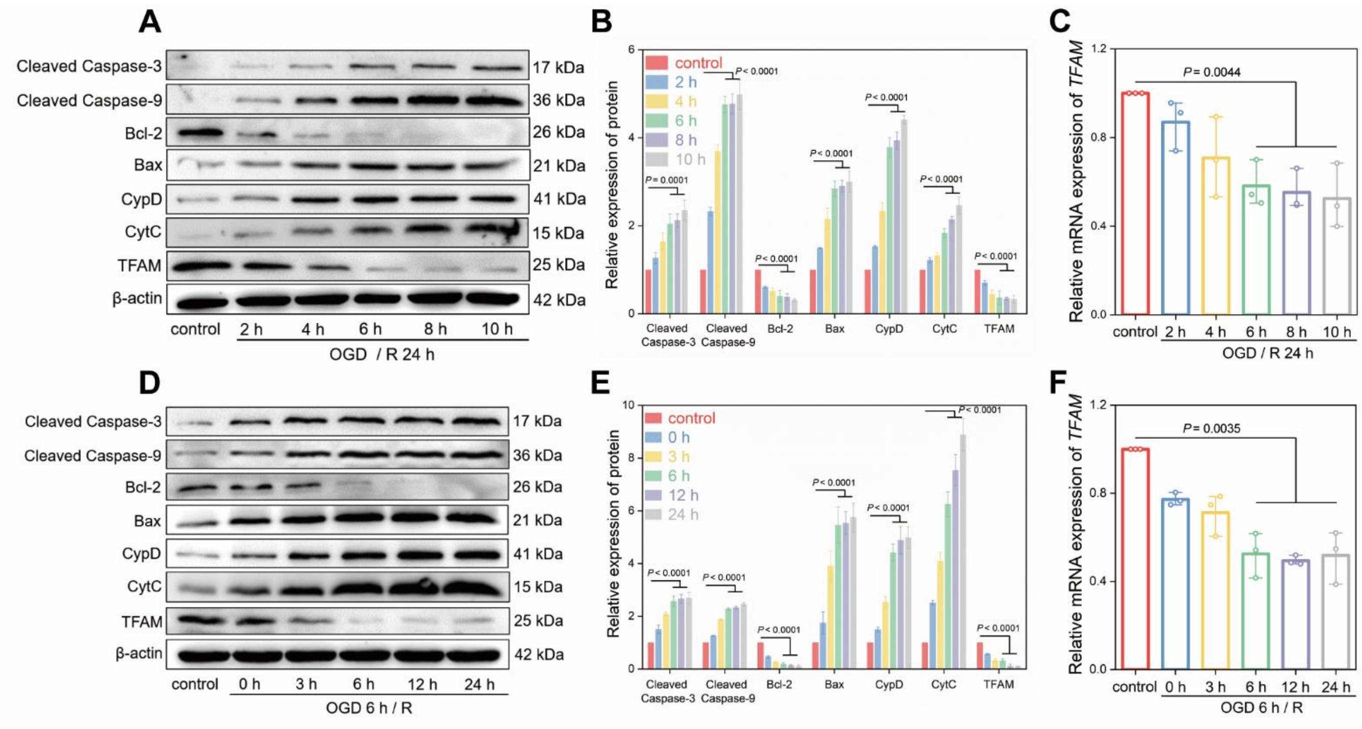

Figure S5 Selection of the optimal time of "Oxygen-Glucose Deprivation/Reoxygenation" (OGD/R). (**A**) Western blot of apoptosis-related protein and TFAM expressions in LO2 cells under OGD for 2, 4, 6, 8, and 10 h, followed by 24 h reoxygenation for the applied time together with quantification (**B**). (**C**) Expression of *TFAM* mRNA tested by qRT-PCR. (**D**) Western blot of apoptosis-related protein and TFAM expressions in LO2 cells under OGD for 6 h, followed by 3, 6, 12, and 24 h reoxygenation for the applied time together with quantification (**E**). (**F**) Expression of *TFAM* mRNA tested by qRT-PCR. Data are expressed as mean ± S.D. (n = 3), (non-repeated ANOVA followed by Tukey's test).

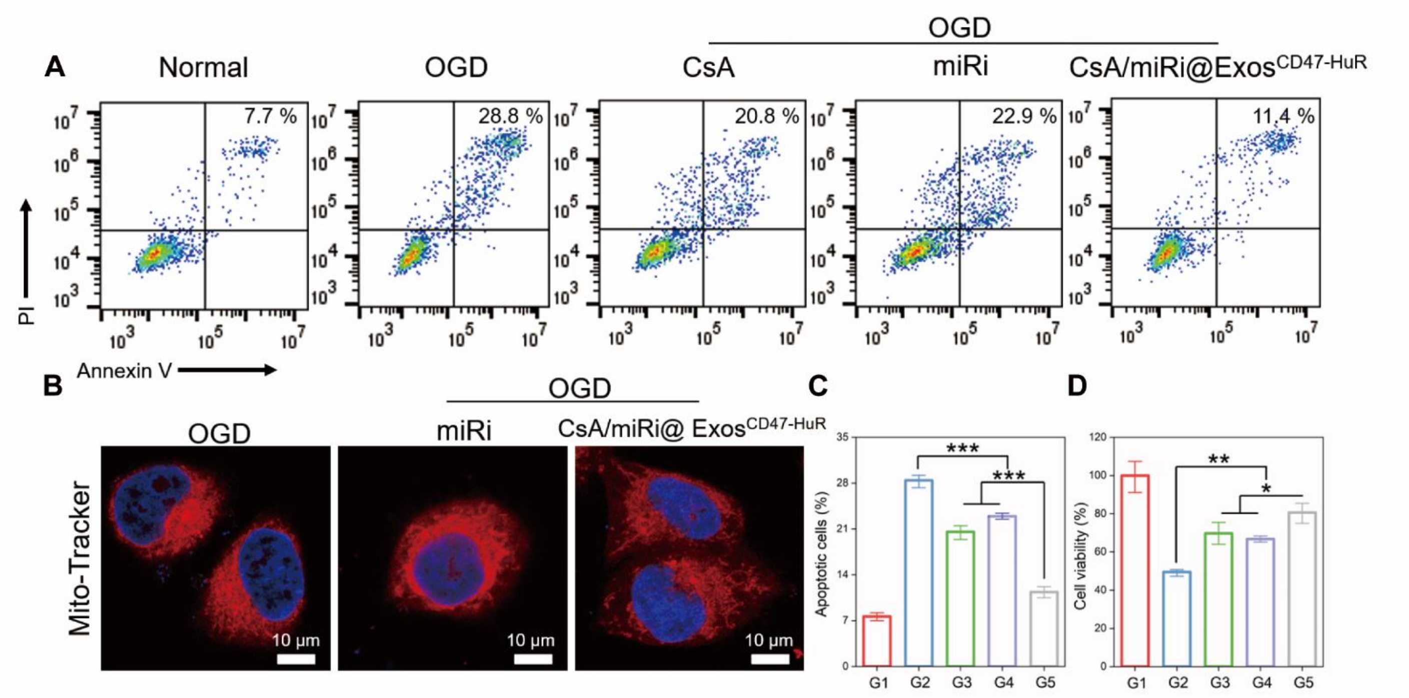

Figure S6 Antiapoptic effects of CsA and miRi in vitro. (**A**) LO2 cell apoptosis was analyzed using Annexin V/PI staining by flow cytometry, with quantitative analysis shown in (**C**). (**B**) Representative CLSM images showing mitochondrial morphology stained with Mito-Tracker (red) and DAPI (blue). Scale bar: 10 μm. (**D**) Cell viability of LO2 cell. Groups were designed as: normal LO2 cells (G1); LO2 cells after OGD/R (G2); LO2 cells pretreated with CsA before OGD/R (G3); LO2 cells pretreated with miRi before OGD/R (G4); LO2 cells pretreated with CsA/miRi@Exos$^{CD47-HuR}$ before OGD/R (G5). miRi refers to hsa-miR-590-3p inhibitor. Data are expressed as mean ± S.D. (n = 3), ****P* < 0.001, ***P* < 0.01, **P* < 0.05, (non-repeated ANOVA followed by Tukey's test).

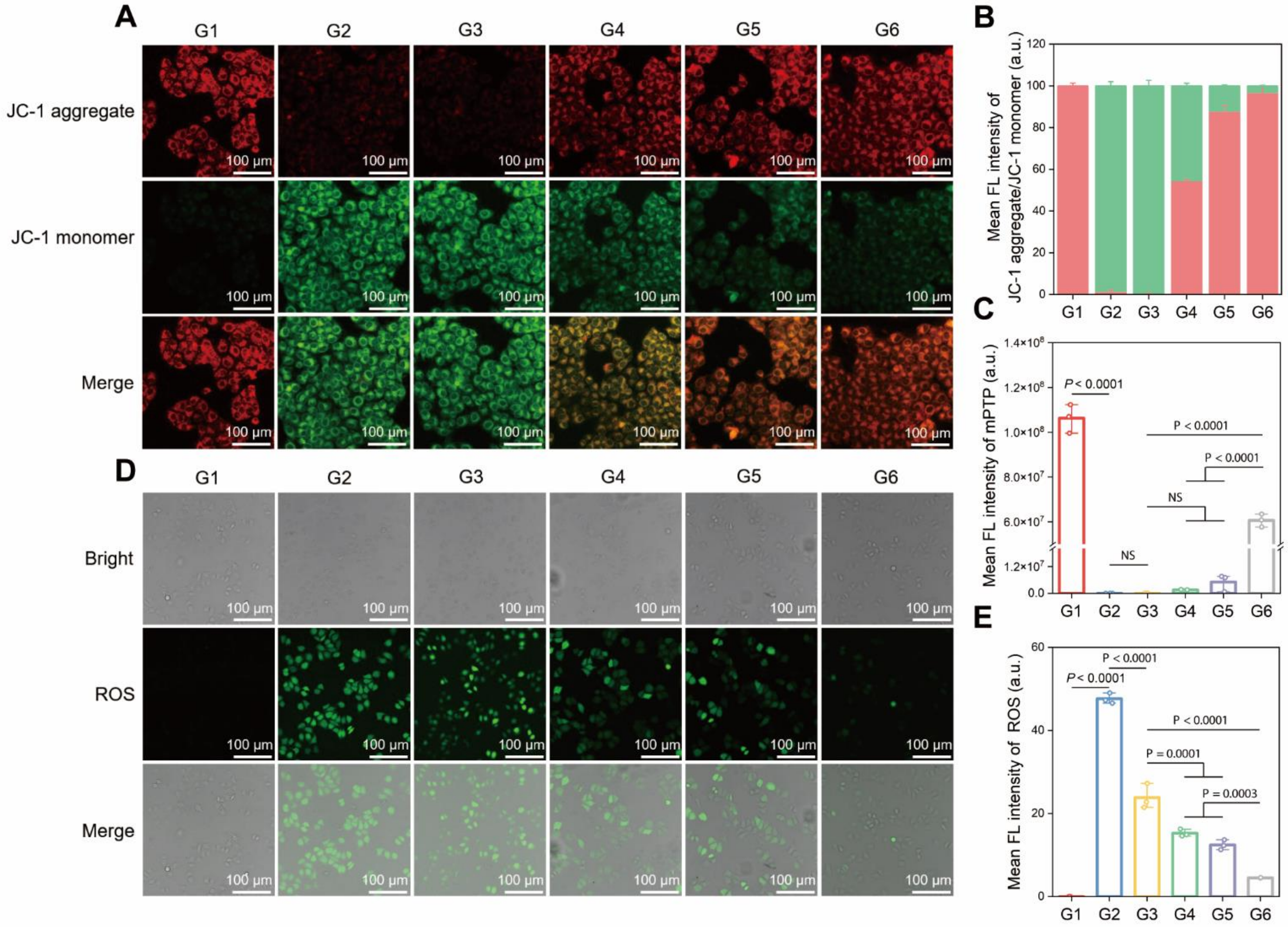

Figure S7 Protection properties in elevating mitochondrial membrane potential (Δψm) and scavenging ROS of CsA/miRi@Exos$^{CD47\text{-}HuR}$ in vitro. (**A**) Δψm analysis of LO2 cells after different treatments, using JC-1 stain. Green fluorescence exhibited JC-1 monomer in cytoplasm and red fluorescence exhibited JC-1 aggregates in core mitochondria. Scale bar: 100 μm. (**B**) Quantitative analysis of fluorescence intensity of JC-1 monomer and JC-1 aggregates, respectively. (**C**) Quantitative analysis of the mPTP opening stained with Calcein AM (green). (**D**) Representative confocal images showing reactive oxygen species (ROS) stained with DCFH-DA (green) together with its fluorescence intensity quantification (**E**). Scale bar: 100 μm. Groups were designed as: normal LO2 cells (G1); LO2 cells after OGD/R (G2); LO2 cells pretreated with Exos$^{CD47\text{-}HuR}$ before OGD/R (G3); LO2 cells pretreated with CsA@Exos$^{CD47\text{-}HuR}$ before OGD/R (G4); LO2 cells pretreated with miRi@Exos$^{CD47\text{-}HuR}$ before OGD/R (G5); LO2 cells pretreated with CsA/miRi@Exos$^{CD47\text{-}HuR}$ before OGD/R (G6). miRi refers to hsa-miR-590-3p inhibitor. Data are expressed as mean ± S.D. (n = 3), NS means no difference (non-repeated ANOVA followed by Tukey's test).

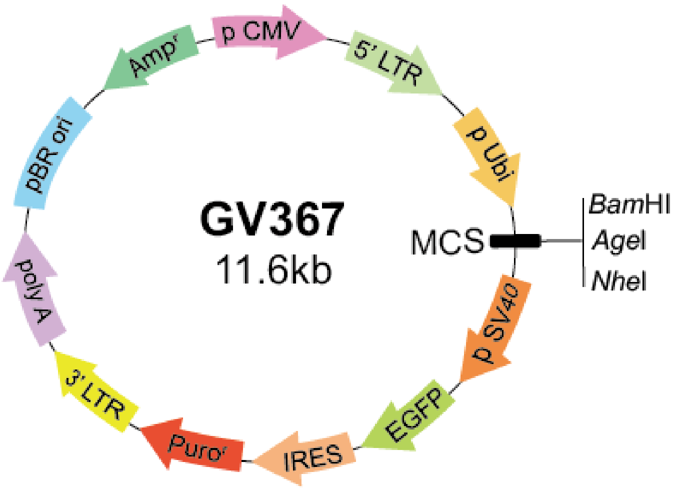

Figure S8 Schematic map of the GV367 lentiviral vector. The vector contains the Ubi promoter (Ubi), multiple cloning site (MCS), SV40 poly(A) signal (SV40), enhanced green fluorescent protein (EGFP) reporter, and puromycin resistance marker (puromycin). The fusion gene was inserted between the AgeI and NheI cloning sites. The construct was verified by sequencing and assigned the control number CON238.

Supplementary Tables

Table S1 The sequences of PCR primer of CD47-HuR fusion gene.

ID	Sequence
ELAVL1(65126-2)-p1	GAGGATCCCCGGGTACCGGTCGCCACCATGTGGCCCCTGGTAGCGGC
ELAVL1(65126-2)-p2	CACACATTCCACAGGCTAGCTTATTTGTGGGACTTGTTGGTTTTGAAGGAAAC

Table S2 Sequences of the miRNA mimics and inhibitors used in the study.

Sequence Name	Sequence (5'-3')
miR-590-3p (hsa)-mimic-sense	UAGCUUAUACAUAAAAUUAUU
miR-590-3p (hsa)-mimic-antisense	UAGCUUAUACAUAAAAUUAUU
miR-590-3p (hsa)-inhibitor	ACUAGCUUAUACAUAAAAUUA
miR-7057-3p (mmu)-mimic-sense	UAAUUUGAUGUCUUUCUCCACC
miR-7057-3p (mmu)-mimic-antisense	UGGAGAAAGACAUCAAAUUAUU
miR-7057-3p (mmu)-inhibitor	GGUGGAGAAAGACAUCAAAUUA

hsa: Homo sapiens; mmu: Mus musculus.

Table S3 Western blot antibodies information.

Antibody	Cat No	Company	Country	Concentration
β-actin	BM3873	Boster	China	1:1000
TFAM	22586-1-AP	proteintech	USA	1:1000
TSG101	28283-1-AP	proteintech	USA	1:1000
CD9	20597-1-AP	proteintech	USA	1:1000
Calnexin	10427-2-AP	proteintech	USA	1:1000
CD81	66866-1-Ig	proteintech	USA	1:1000
Cleaved caspase 3	A11021	abclonal	China	1:1000
Cleaved caspase 9	A22673	abclonal	China	1:1000
Bcl-2	WL01556	Wanlei	China	1:500
Bax	50599-2-Ig	proteintech	USA	1:1000
CytC	10993-1-AP	proteintech	USA	1:1000
CypD	12716-1-AP	proteintech	USA	1:1000
Bcl-2	WL01556	Wanlei	China	1:500
F4/80	A1256	abclonal	China	1:1000
HRP-conjugated goat anti-rabbit secondary antibody	BM2006	Boster	China	1:2000
HRP-conjugated goat anti-mouse secondary antibody	BA1051	Boster	China	1:2000

Table S4 Primers for qRT-PCR used in the study.

Gene	Forward (5’-3’)	Reverse (5’-3’)
β-actin	CCTGGCACCCAGCACAAT	GGGCCGGACTCGTCATAC
TFAM	CGCTCCCCCTTCAGTTTTGT	CCAACGCTGGGCAATTCTTC

Table S5 Suzuki score for the assessment of liver damage following hepatic ischemia/reperfusion

Score	Congestion	Cytoplasmic vacuolization	Parenchymal necrosis
0	No	No	No
1	Minimal	Minimal	Single-cell necrosis
2	Mild	Mild	＜ 30%
3	Moderate	Moderate	＜ 60%
4	Severe	Severe	＞ 60%
